# Design principles of gene evolution for niche adaptation through changes in protein–protein interaction networks

**DOI:** 10.1038/s41598-020-71976-x

**Published:** 2020-09-24

**Authors:** Gon Carmi, Somnath Tagore, Alessandro Gorohovski, Aviad Sivan, Dorith Raviv-Shay, Milana Frenkel-Morgenstern

**Affiliations:** 1 The Azrieli Faculty of Medicine, Bar-Ilan University, 8 Henrietta Szold St, 13195 Safed, Israel; 2 Department of Systems Biology, Columbia University Medical Center, Herbert Irving Cancer Research Center, New York, USA

**Keywords:** Computational models, Ecological networks

## Abstract

In contrast to fossorial and above-ground organisms, subterranean species have adapted to the extreme stresses of living underground. We analyzed the predicted protein–protein interactions (PPIs) of all gene products, including those of stress-response genes, among nine subterranean, ten fossorial, and 13 aboveground species. We considered 10,314 unique orthologous protein families and constructed 5,879,879 PPIs in all organisms using ChiPPI. We found strong association between PPI network modulation and adaptation to specific habitats, noting that mutations in genes and changes in protein sequences were not linked directly with niche adaptation in the organisms sampled. Thus, orthologous hypoxia, heat-shock, and circadian clock proteins were found to cluster according to habitat, based on PPIs rather than on sequence similarities. Curiously, "ordered" domains were preserved in aboveground species, while "disordered" domains were conserved in subterranean organisms, and confirmed for proteins in DistProt database. Furthermore, proteins with disordered regions were found to adopt significantly less optimal codon usage in subterranean species than in fossorial and above-ground species. These findings reveal design principles of protein networks by means of alterations in protein domains, thus providing insight into deep mechanisms of evolutionary adaptation, generally, and particularly of species to underground living and other confined habitats.

## Introduction

Subterranean animals represent an excellent model for studying the evolution of adaptation to life underground and its stresses, generally associated with life in confined environments, such as dry- and dump-woods and caves. These animals spend their entire lives below ground. As such, they experience relatively stable fluctuations in temperature and humidity, yet face multiple stresses, such as darkness, hypoxia, hypercapnia (high levels of carbon dioxide), and multiple pathogens^1–4^. Fossorial animals inhabit both underground and aboveground habitats, with varying amounts of time spent in each^5–7^. Thus, comparing subterranean animals with fossorial and aboveground animals offers a prime opportunity for studying evolution in the face of environmental stresses^8,9^. Although underground-dwelling organisms have been extensively studied^1,2,10–12^, the evolution of their cellular networks and protein–protein interactions (PPIs), particularly those involving stress response genes, remains elusive. While extreme changes in habitat may affect protein sequence, structure, and function, the impact of such changes on corresponding cellular networks has not been studied in detail. According to the domain-oriented view, proteins are built from a set of domains corresponding to conserved regions with distinct functional and structural characteristics^13–15^. As might be expected, rearranged domain combinations (via exon shuffling or mixing) may result in the emergence of new PPI networks (as occurred during metazoan evolution). The evolutionary pressure of niche adaptation is assumed to act upon random changes in gene expression. Here, we considered an alternative view whereby functional properties of proteins within defined PPI networks can be direct selected by such evolutionary pressure.

Our previously developed ChiPPI^15^ predictive tool is based on the integration of true PPI data from BioGrid (release 3.4.163)^16^, a database of experimentally verified PPIs, and the protein domain content of the interacting proteins. ChiPPI utilizes protein domains of interacting proteins to predict interactions of orthologous proteins. In the current study, we used ChiPPI to identify changes in PPI networks that occur upon switching protein domains within otherwise conserved orthologous proteins. Accordingly, we found that every change in protein sequence or domain content in an orthologous protein throughout evolution resulted in advantageous addition to or disruption of a PPI network. The ChiPPI tool is designed to interpret and represent such changes as alterations in PPI networks. We thus predicted all PPI networks of 32 species living in three broad ecological niches, namely, subterranean (terrestrial and aquatic caves, woods and underground), fossorial, and aboveground (terrestrial and aquatic niches, such as rivers) habitats.

Our efforts revealed that the functional expression of genetic change is mostly associated with changes in PPI networks as species adapt to a new niche, rather than with changes in protein sequences. Since niche adaptation likely requires changes in cellular functions that regulates heat, oxygen, carbon dioxide levels, and light, we studied the PPI networks of the relevant stress response proteins using ChiPPI. Our findings infer that organisms adapt to their environment largely by species-specific alterations in PPI networks, and by "shuffling" (or "mixing") protein domains, rather than by point sequence mutations. Orthologous hypoxia, heat-shock, and circadian clock proteins were found to cluster according to their corresponding broad ecological niches (i.e., subterranean, fossorial or aboveground), based on PPI conservation, rather than by protein sequence conservation. Interestingly, we found that over the course of evolution, "ordered" domains (domains with defined 2-dimension (2D) or 3D structure) were preserved in aboveground species, while "disordered" domains were conserved in subterranean organisms. Moreover, we found that genes encoding proteins with disordered regions presented adapted non-optimal codon usage. Accordingly, such proteins form at least 35% fewer PPIs than do abundant proteins with ordered and mixed regions. Furthermore, subterranean proteins have at least 14% significantly lower codon usage preference scores than do animals from the other habitats. Thus, we demonstrated that the evolution-driven “ordered” domains of aboveground species adapted to include more connected networks than did domains in the homologous proteins of subterranean species. These findings highlight the complicated adaptation process based on protein networks rather than point mutations, as described frequently in evolutionary studies.

## Results

### Data collection

**Figure 1:**
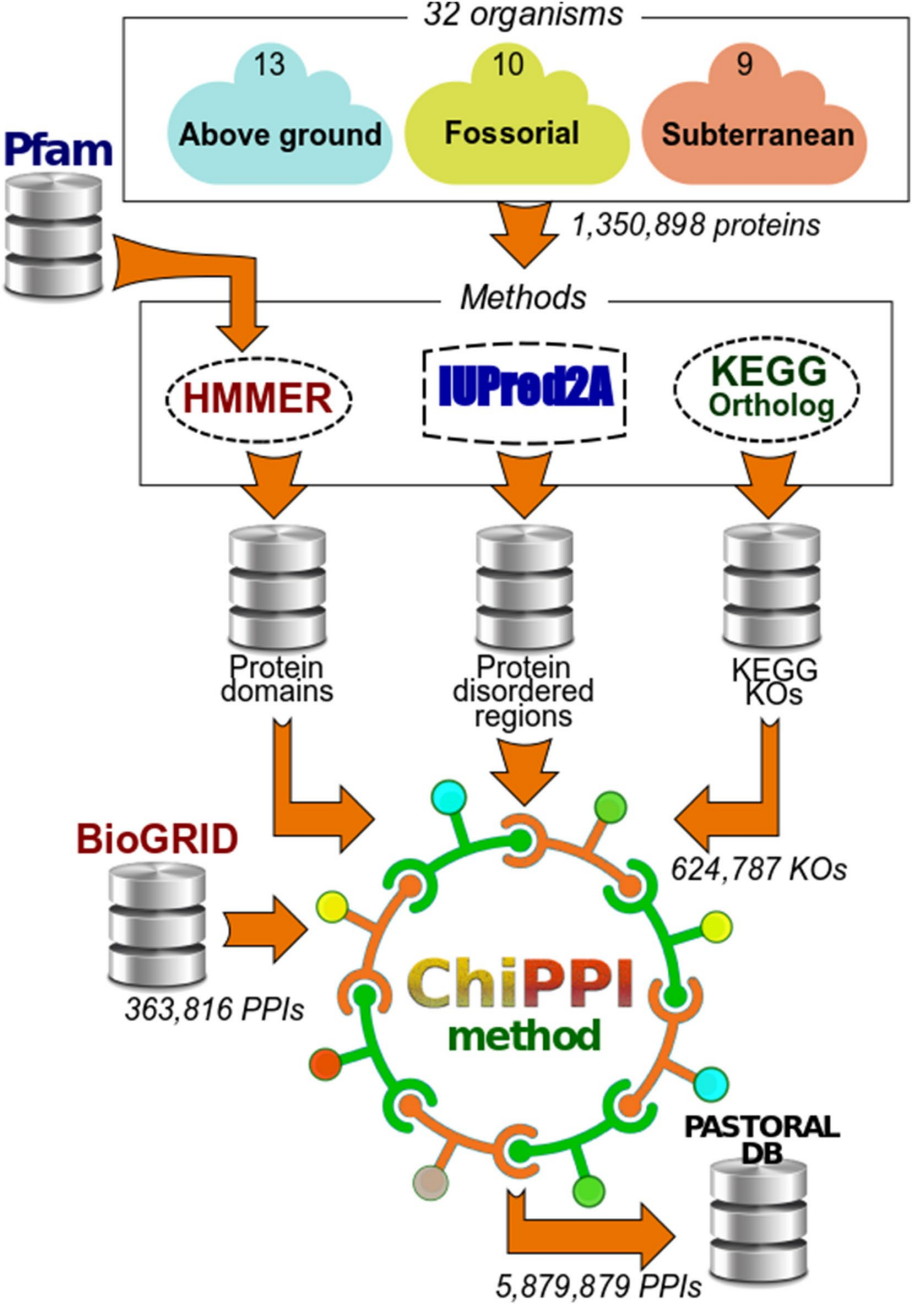
The study overview. Fully sequenced genomes with coding sequences and annotated proteomes were collected from 32 species living in three broad ecological niches: subterranean, fossorial, and aboveground. For collected proteins (1,350,898), protein domains, protein disordered regions, and KEGG orthologous annotation (624,787) were predicted using the Pfam search tool^53^ along with HMMER^60^ , IUPred2A^44^, and the KEGG database^17^, respectively. Next, 5,879,879 PPIs were evaluated using our previously developed ChiPPI tool^15^. Briefly, ChiPPI uses a domain-domain co-occurrence table. When a certain domain is missing, ChiPPI evaluates the corresponding missing interactors in the PPI network^15^, based on real PPI data (363,816) as obtained from BioGrid (release 3.4.163)^16^. Finally, PPI data are organized in PASTORAL, a dedicated database.

We hypothesized that the evolution of underground species affected protein networks in a unique manner in which various types of protein domains served as building blocks of protein evolution. To study the evolution of protein networks, we collected genomic, proteomic, and protein domain classification data, namely, fully sequenced genomes with coding sequences and annotated proteomes, together with protein ortholog assignments, from 32 species living in three broad ecological niches, namely subterranean, fossorial, and aboveground (Table 1, and listed in Materials and Methods). We first sought overall statistics regarding the number of proteins and the number of corresponding orthologous protein families. Overall PPI statistics were calculated, including those predicting PPIs in organisms for which experimentally verified PPI data are missing. We used the KEGG orthologs (KO) group of orthologous proteins in KEGG (Kyoto Encyclopaedia of Genes and Genomes)^17^ to reproduce gain and loss of protein domains in orthologous proteins. We collected 1,350,898 proteins from the studied organisms that belong to 624,787 KO groups (10,314 are unique ortholog groups). The matching number of interactors and networks for every organism were exhaustively calculated for all these proteins (Fig. 1). We found that 361,615 of the 1,350,898 proteins are distributed among 5,879,879 (predicted and real) PPIs. The mean number of interactors per protein within each habitat, namely, aboveground (A), fossorial (F), and subterranean (S) were 32.07, 32.48, and 32.67, respectively (see details in the supplementary results and in Tables S1–S3). This shows that the number of interactors per protein is similar for organisms from different ecologies.

**Table 1:** All organisms included in the PASTORAL database, with a complete number of proteins in the corresponding proteome.

Ecology	Organism Name	Organism ID	Heat-shock		Hypoxia-related		Circadian	
			Proteins	PPIs	Proteins	PPIs	Proteins	PPIs
F	*Condylura cristata*	COC	17	2,325	9	436	1	37
F	*Camponotus floridanus*	cfo	9	1,421	2	205	2	55
F	*Manis javanica*	mjv	62	4,577	10	321	8	379
F	*Solenopsis Invicta*	soc	9	1,325	1	47	2	55
F	*Cricetulus griseus*	cge	9	1,994	6	273	3	53
F	*Dasypus novemcinctus*	DAN	19	2,433	9	439	2	37
F	*Dipodomys ordii*	DIO	18	2,431	9	443	3	50
F	*Microtus ochrogaster*	MIO	20	2,586	9	463	3	57
F	*Octodon degus*	OCD	20	2,552	9	466	3	56
F	*Peromyscus maniculatus bairdii*	PEM	19	2,504	9	452	3	58
S	*Astyanax mexicanus*	ASM	40	3,975	6	295	8	364
S	*Cryptotermes secundus*	CRS	8	1,248	2	65	1	36
S	*Folsomia candida*	fcd	9	1,323	5	313	1	34
S	*Myotis lucifugus*	MYL	63	4,846	9	340	8	380
S	*Zootermopsis nevadensis*	zne	10	1,691	2	70	2	48
S	*Chrysochloris asiatica*	CHA	20	2,402	9	472	2	44
S	*Fukomys damarensis*	FUD	20	2,408	9	442	2	42
S	*Heterocephalus glaber*	hgl	19	2,572	8	311	4	62
S	*Nannospalax galili*	ngi	21	2,603	9	462	4	62
A	*Bos taurus*	bta	18	1,512	9	301	4	55
A	*Drosophila melanogaster*	dme	15	486	3	30	2	10
A	*Danio rerio*	dre	47	5,137	9	375	8	404
A	*Erinaceus europaeus*	ERE	30	2,293	16	424	4	61
A	*Felis catus*	fca	16	1,416	8	235	3	49
A	*Gallus gallus*	gga	17	1,447	8	260	4	47
A	*Homo sapiens*	hsa	35	2,850	10	484	5	98
A	*Mus musculus*	mmu	32	1,672	9	306	6	61
A	*Ornithorhynchus anatinus*	oaa	13	1,123	7	234	2	23
A	*Orycteropus afer afer*	ORA	35	2,115	10	427	4	78
A	*Pan troglodytes*	ptr	19	1,486	10	309	5	65
A	*Rattus norvegicus*	rno	22	1,668	7	276	5	62
A	*Sus scrofa*	ssc	19	1,519	16	280	5	55

The number of heat-shock proteins, hypoxia-related proteins and circadian proteins, along with their PPIs, was assessed and collected in PASTORAL. Organisms in KEGG are coded by lowercase letters; organisms not in KEGG are coded by uppercase letters. A-aboveground, F-fossorial, and S-subterranean organisms.

Additional analysis of PPI features for orthologous proteins (516 KOs) common to all organisms were similar across ecologies. These features included the number of interactors, the number of PPIs, and global/individual clustering coefficients (supplementary results, Figures S1, S2, Table S4). Thus, we studied PPI properties of genes encoding products related to stresses that differ across the ecologies considered, such as hypoxia. Our findings confirm our hypothesis that the design principles of the evolution of underground species involve various types of protein domains serving as building blocks of protein evolution.

### Analysis of the PPIs of stress-response proteins cluster organisms according to habitat

To examine how organisms might have adapted to the various stresses in each habitat, we analyzed mutations and changes in the PPIs encoded by stress response genes. Heat-shock, hypoxia, and circadian stresses differ considerably between aboveground and underground environments, and are likely to drive evolutionary selection of proteins that provide optimal function in each niche^1,9^. We assumed that organisms subject to a shared ecological experience would face similar environmental stresses. PPI networks of stress-related proteins would thus be expected to differ substantially according to ecology.

To test our hypothesis, we performed clustering analysis of all the organisms included in our study, based on mutations and PPI network features, and compared the results for each classification. Such analysis included all orthologous stress-response, hypoxia, heat-shock, and circadian stress proteins (Table 1). In total, 85,173 PPIs related to stress-response proteins were found to be distributed among 1,103 proteins. These comprised of 730 heat shock proteins in 71,940 PPIs, 254 hypoxia-related proteins in 10,256 PPIs, and 119 circadian proteins in 2,977 PPIs (Table 1, Tables S1–S7). All orthologous stress-response genes (KO groups) were obtained by querying the KEGG database with the terms “heat-shock”, “hypoxia”, and “circadian” terms. The results are listed in Table 2, while the corresponding lists of proteins are found in Tables S5, S6 and S7, respectively.

**Table 2:** KEGG Orthologs: Heat-shock (upper), hypoxia-related (middle) and circadian (bottom) proteins.

KO	Info
19369	HSPB11; heat shock protein beta-11
19765	HSBP1; heat shock factor-binding protein 1
3283	HSPA1_8; heat shock 70 kDa protein 1/8
4455	HSPB1; heat shock protein beta-1
8879	HSPB8; heat shock protein beta-8
9414	HSF1; heat shock transcription factor 1
9415	HSF2; heat shock transcription factor 2
9416	HSF3; heat shock transcription factor 3
9417	HSF4; heat shock transcription factor 4
9485	HSP110; heat shock protein 110 kDa
9487	HSP90B, TRA1; heat shock protein 90 kDa beta
9489	HSPA4; heat shock 70 kDa protein 4
9490	HSPA5, BIP; heat shock 70 kDa protein 5
9543	HSPB2; heat shock protein beta-2
9544	HSPB3; heat shock protein beta-3
9545	HSPB6; heat shock protein beta-6
9546	HSPB7; heat shock protein beta-7
9547	HSPB9; heat shock protein beta-9
18055	HIF1AN; hypoxia-inducible factor 1-alpha inhibitor (HIF hydroxylase)
6711	PH-4; hypoxia-inducible factor prolyl 4-hydroxylase
8268	HIF1A; hypoxia-inducible factor 1 alpha
9095	HIF2A, EPAS1; hypoxia-inducible factor 2 alpha
9096	HIF3A; hypoxia-inducible factor 3 alpha
9486	HYOU1; hypoxia up-regulated 1
9592	EGLN, HPH; hypoxia-inducible factor prolyl hydroxylase
2223	CLOCK, KAT13D; circadian locomotor output cycles kaput protein [EC:2.3.1.48]
2633	PER2; period circadian protein 2
21599	CIART, CHRONO, GM129; circadian-associated transcriptional repressor
21753	PASD1; circadian clock protein PASD1
21944	PER1; period circadian protein 1
21945	PER3; period circadian protein 3

**Figure 2:**
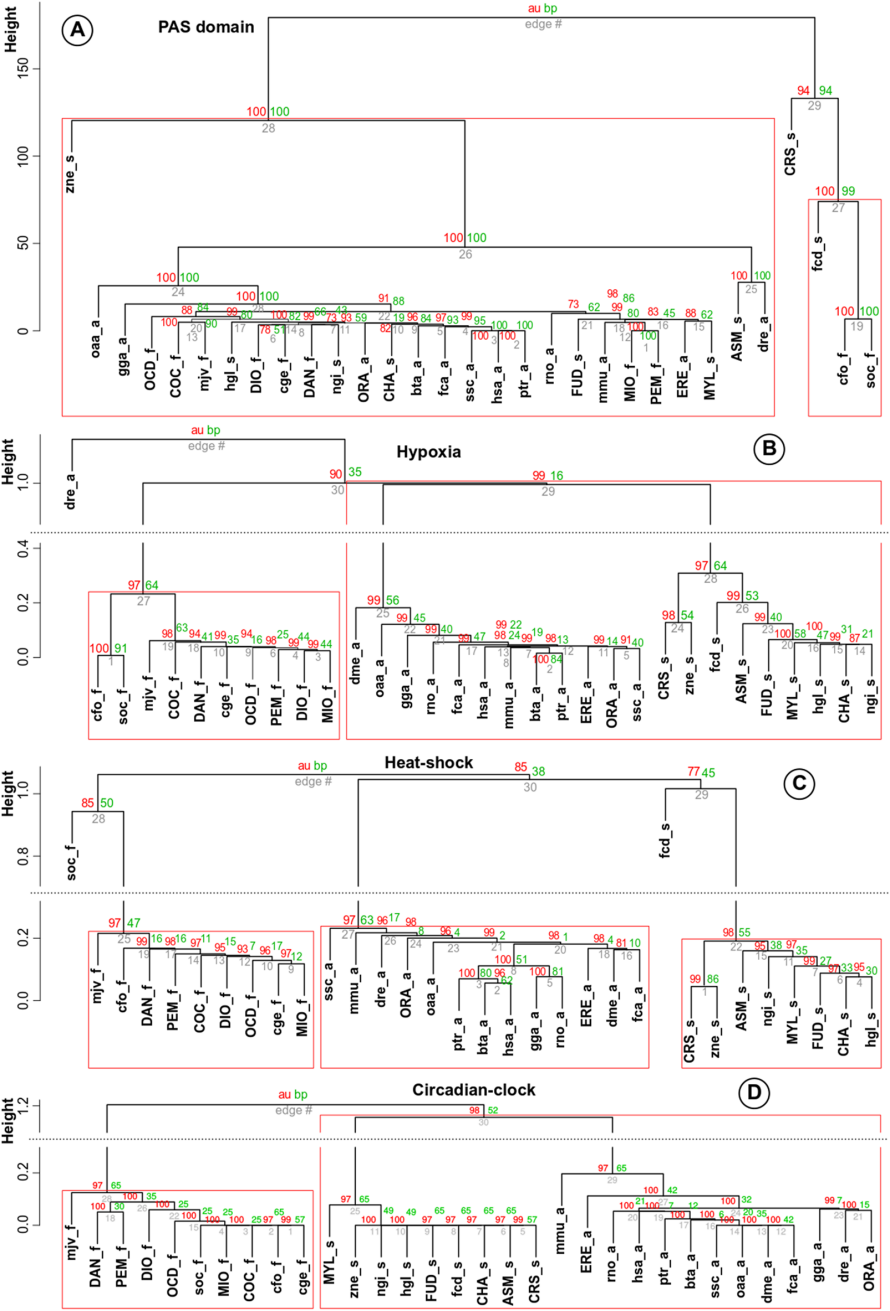
Hierarchical clustering. (**A**) All studied organisms were assessed for hierarchal clustering using an identity matrix from multiple alignment of PAS domain sequences from hypoxia-related orthologous proteins. (**B**) Clustering of the hypoxia-related proteins (in the 32 organisms studied (Table 2). (**C**) Clustering of the heat-shock proteins (Table 2). (**D**) Clustering of circadian proteins (Table 2). AU (approximately unbiased) *p* values and BP (bBootstrap probability) values are shown^59^.

Next, we performed clustering analysis based on sequence mutations and PPI features for the full set of heat-shock, hypoxia, and circadian stress proteins (Table 2). Remarkably, proteins related to hypoxia, heat-shock, and circadian stresses in the 32 organisms studied did not all cluster according to shared ecology based on sequence mutations (Fig. 2A) but significantly did so on the basis of "PPI network clustering coefficient" (Fig. 2B–D; *p* value (AU) < 0.02, *p* value = 0.0018, and *p* value = 0.0013, respectively, Pearson's χ^2^-test). Moreover, the observed clustering of organisms according to ecological niches reflects adaptation towards a specific stress, rather than to the particular identity of the environment, such as a cave or within soil. Interestingly, we observed that bat clustered with other subterranean organisms based on hypoxia-related proteins. As hypoxia has been associated with spill-over, i.e., transmission of virulent viruses to other species^18^, other subterranean organisms may also have innate protection from virulent viruses. Moreover, the little brown bat (*Myotis lucifugus*) is associated with the emergence of SARS-CoV-2 responsible for the current COVID-19 pandemic^19^. Additional contributors to the spill-over of virulent viruses from bats include arousal from hibernation and the fact that hundreds of these bats hibernate in caves^18,20^. Taken together, these results showed better assignment of organisms to broad ecological niches based on their cellular PPI networks than on sequence mutations, and supports the hypothesis that organisms adapt to their specific ecologies by modulating PPI networks rather than by mutation of protein sequences.

Additional analysis of PPI networks involving hypoxia-related proteins (e.g. HIF2A) revealed that distribution of central proteins within PPI network discriminates between PPIs of different ecologies, such as DMAD3, XPO1 and EWSR1, were unique to subterranean animals (supplementary results, Figure S3). This finding indicates that adaption to ecology via PPI modulation could rely on “shuffling” of protein domains, resulting in global changes in PPI networks in an ecology-specific manner.

### Genes encoding common orthologous proteins of subterranean animals adopted non-optimal codons

Due to redundancy of the genetic code, amino acids are encoded by multiple synonymous codons. Moreover, the use of synonymous codons is non-uniform, such that there is a strong preference for certain codons in highly expressed genes^21–23^. According to the strength of affinity of codon-anticodon interactions, codons with high and low affinities are referred to as optimal and non-optimal, respectively^24,25^. We previously showed that subterranean animals adopted non-optimal codon usage as part of their adaptation to their stressful environment^26^. We now hypothesized that orthologous proteins of subterranean animals adopted different codon usage preferences than those of fossorial and aboveground species.

**Figure 3:**
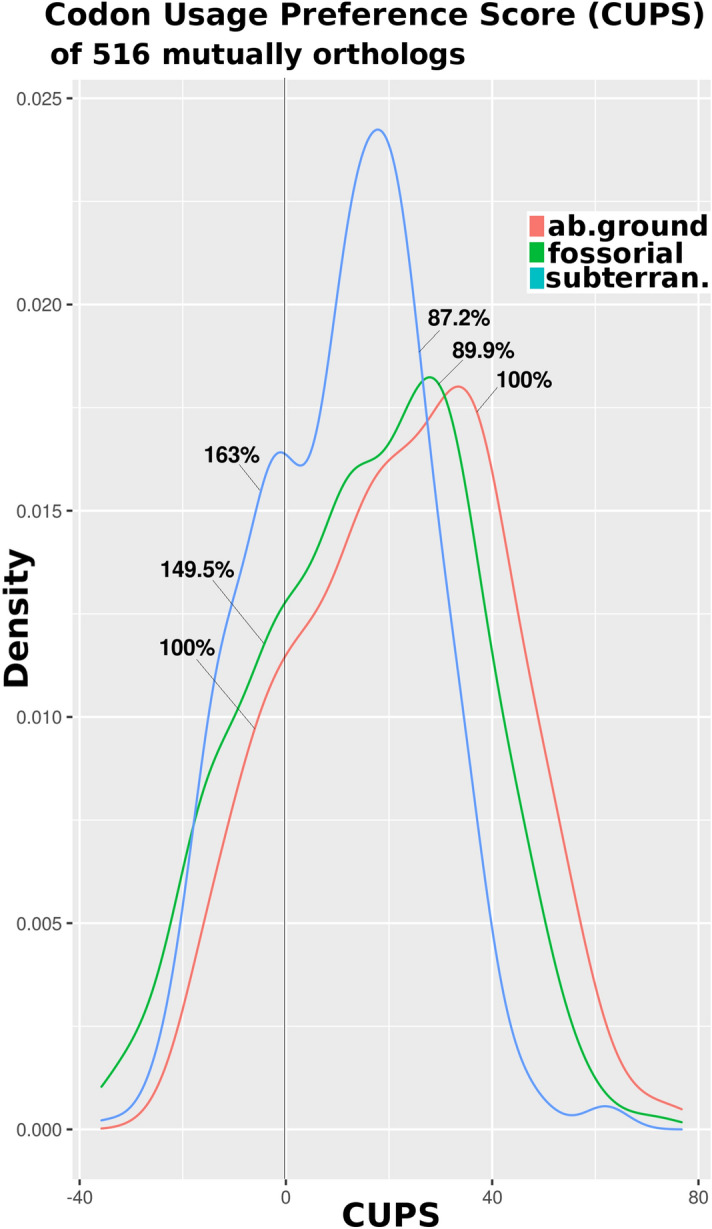
Codon usage preference score (CUPS) density plots for 516 KOs common to all studied organisms. (**A**) Density plot for all 516 KOs in each habitat (ab.ground (aboveground), fossorial, and subterranean). Proportions (%) of KOs, relative to those seen in aboveground dwellers are indicated in terms of optimal (positive) and non-optimal (negative) codon usage presences (CUPS). Subterranean organisms adopt 75.0% (*p* value = 0.0008) more non-optimal codon usage than do aboveground species.

To examine differences in codon usage preferences, we considered 516 orthologous proteins from KO groups common to the 32 organisms of study by developing a tendency score to estimate codon usage preferences (codon usage preference score (CUPS), defined by Eq. (3)) from a codon usage table (CUT). Accordingly, we classified codons as optimal and non-optimal^25^. For the 516 common KO proteins, we computed the probability of subterranean animals adopting non-optimal and optimal codon usage, as calculated from the area under the density distribution curve, relative to aboveground animals (Fig. 3). Using the bootstrapping procedure described below, we found that subterranean and fossorial animals adopted 75.0% (*p* value = 0.0019) and 58.8% (*p* value = 0.076) more non-optimal codon usage, respectively, compared with aboveground animals (Fig. 3). Briefly, 10,000 random groups of 516 KOs were generated (as bootstrap replicates) and codon usage was calculated. *p* values were defined as the frequency of bootstrap replicates, with calculated values equaling or exceeding observed values (see Materials and Methods). We found that subterranean animals adopted 50.05%, on average, less optimal codon usage (CUPS: (subterranean (S) = 11.20, aboveground (A) = 22.42, A vs. S, *p* value < 2.2 × 10^–16^. Wilcoxon rank sum test with continuity correction; Table S4).

### Proteins with disordered regions are encoded by genes that adopted non-optimal codon usage and form fewer PPIs

Traditionally, proteins realize their function based on their 3-dimensional structure. However, in recent years, protein segments (> 30 residues) lacking stable secondary and/or tertiary structure, referred to as intrinsically disordered regions (IDRs) or intrinsically disordered protein regions (IDPRs), have been shown to exhibit functional capabilities within core molecular processes^27–32^. The tendency of a protein region to exhibit structure can be represented on a spectrum^33^. At one extreme, proteins without IDRs are considered as structured, while at the other end, proteins without structure over the entire sequence are referred to as intrinsically disordered proteins (IDPs)^27–32^. Differential inclusion of IDRs via alternative splicing was found to increase protein function capabilities. IDRs contain sequence motifs which mediate interactions, and can contain post-translational modification sites^34–36^. Differential inclusion of IDRs was also found to modulate PPIs in an tissue-specific manner by including or excluding IDRs that interact directly with protein partners^34,35^. IDR composition, length and position were, moreover, shown to affect protein half-life, in addition to expanding protein functional capabilities^37–40^. Misregulation and mutations within IDRs affect molecular function^41–43^. The presence of a high proportion of missense disease mutations within IDRs indicates the importance of IDRs to proper molecular function, as well as to the development of disease. Therefore, we expanded the 516 KO groups common to all organisms addressed in this study to consider all KO groups and intrinsically disordered regions in proteins, defined as a continuous stretch longer than seven residues with an IUPRED SCORE >  = 0.5^44^ that do not overlap with Pfam domains. We thus hypothesized that disordered segments would affect ecological adaptation; and examined this by systematic analysis of multiple data sets that describe the sequences of various ordered and disordered domains, as well as proteins with both ordered and disordered regions, roughly corresponding to structured proteins, IDPs and IDRs respectively.

**Figure 4:**
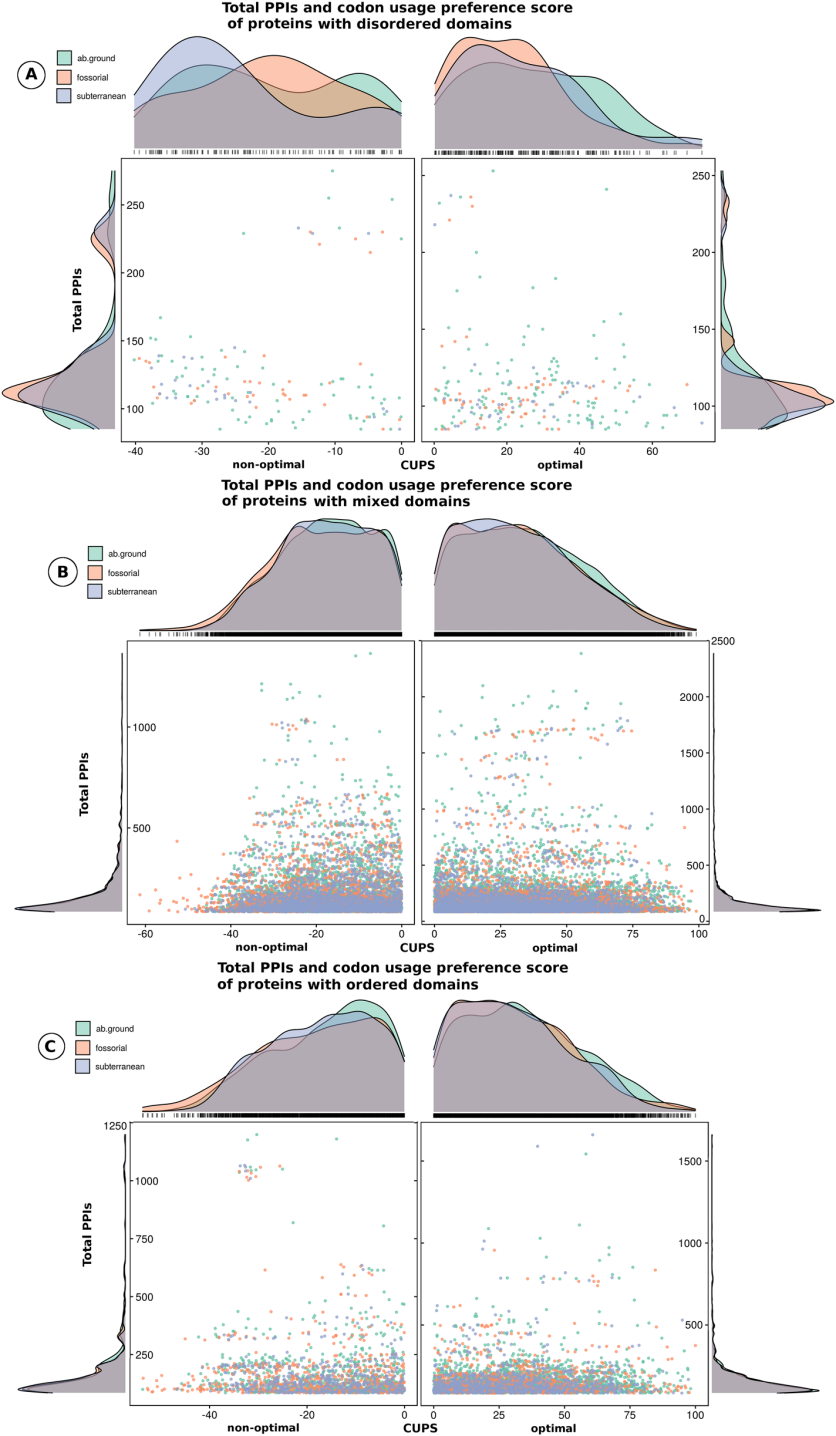
Total PPIs and codon usage preference score (CUPS) with density plots for all proteins with disordered (**A**), mixed (**B**), and ordered (**C**) regions, stratified by ecology (aboveground, lime green (#66c2a5) *; fossorial, soft orange (#fc8d62) *; and subterranean, light blue (#8da0cb)*). Proteins with disordered, mixed, and ordered regions formed different clusters, with varying degrees of extreme values of PPIs and CUPS. Additionally, proteins with disordered regions that adopted non-optimal CUPS [− 50, − 20] were more numerous for subterranean animals than for their counterpart proteins in fossorial and aboveground animals. *Hexadecimal color number.

Once again, we calculated the total number of PPIs and CUPS and generated scatter plots (Fig. 4). These plots were generated from orthologous proteins, with the total number of PPIs differing significantly, at least by 1.2-fold, between ecologies. We found that proteins with disordered regions generally form fewer PPIs and are encoded by more genes showing non-optimal codon usage preferences to higher degree (Fig. 4A), relative to their counterparts containing mixed (Fig. 4B) and ordered (Fig. 4C) regions. On average, proteins with disordered regions formed 35.2%, 36.92%, and 35.6% fewer PPIs than did proteins with ordered regions within aboveground, fossorial, and subterranean ecologies, respectively (*p* value < 2.2e−16, Wilcoxon rank sum test with continuity correction;Table S2). Moreover, proteins with disordered regions adopt, on average, 11.2%, 12.8%, and 7.6% less optimal codon usage (CUPS) than do proteins with mixed regions from aboveground, fossorial, and subterranean ecologies, respectively (*p* value < 0.024, Wilcoxon rank sum test with continuity correction, Table S8). These results indicate that proteins with disordered regions form fewer PPIs and are encoded by genes that adopted fewer optimal codon usage preferences than do counterpart proteins with ordered and mixed regions.

Collectively, our findings are consistent and extend observations made with the fungus *Neurospora*, namely that non-optimal codons are used more often in intrinsically disordered regions, while optimal codons are preferentially used in structured (ordered) domains^45^. Moreover, experimentally optimizing codon usage of the circadian clock gene was found to impair gene function^45^, thus demonstrating the functional role of IDRs in protein function, in general^32,46^, and the functional role of non-optimal codons, in particular. The results were similar when proteins with disordered regions were compared across ecologies (supplementary results, Tables S2, S8 and S9).

We observed a higher proportion in the mean number of interactors among aboveground than subterranean animals (93.1% (ordered), 97.7% (mixed), and 147.8% (disordered), *p* value 1.15e−11, Pearson's χ^2^-test; Table S8). This result indicates higher connectivity in the PPI networks of aboveground animals. Additionally, PPIs in subterranean, fossorial, and aboveground species displayed significant enrichment, compared with the 17,266 instances of loss of protein domains in 10,000 random PPI networks (125,956; 172,613; and 212,941, compared to 81,622 PPIs; V = 52, *p* value = 0.009766, V = 40, *p* value = 0.03906, and p = V = 91, *p* value = 0.0002441, Wilcoxon signed rank test with continuity correction), respectively.

These observed interactions involved 9,429; 10,676; and 13,077 proteins, on average, in subterranean, fossorial, and aboveground species (V = 36, *p* value = 0.4316, V = 21, *p* value = 0.9102, V = 89, *p* value = 0.0007324, respectively, Wilcoxon signed rank test with continuity correction), respectively. These values are thus significantly higher than the average 10,000 random PPI networks only for aboveground species. This is possibly due to the low number of proteins in PPIs belonging to fossorial and subterranean insects considered. Indeed, the average numbers of interactors per protein as a function of habitat (i.e., 32.67 (S), 32.48 (F) and 32.07 (A)) were significantly higher compared with the random value (16.5) (V = 55, *p* value = 0.001953, V = 45, *p* value = 0.003906, V = 91, *p* value = 0.0002441, respectively, Wilcoxon signed rank test with continuity correction). To confirm our results regarding codon usage preferences and PPIs, we collected such information from 61 proteins from the DisProt^47–49^ database with over 98% disorder content from *Rattus norvegicus, Mus musculus, Homo sapiens, Drosophila melanogaster, Danio rerio, Sus scrofa and Bos taurus* (Table S10)*.*

Orthologous proteins were found in aboveground, fossorial and subterranean animals, and CUPS and PPI analysis were performed. We found that subterranean animals adopt extreme non-optimal codon usage preferences and form less PPIs that are on average relative to aboveground and fossorial ecologies (CUPS ( PPIs)): − 17.37 (43.57), − 14.71 (45.74) and − 14.76 (44.86) respectively (*p* value < 0.041, Wilcoxon rank sum test with continuity correction, Tables S11, S12, S13, respectively). These patterns are apparent in a scatter plot showing density distributions (Fig. S4). The results replicated the observations obtained from our classification of proteins as ordered, disordered or mixed. Moreover, as this analysis was performed without consideration of our ecology-based classification, our results are independent of our domain classification method. Furthermore, the results confirm that our classification method captures many aspects of the disordered nature of proteins, at least in relation to their adaptation to a subterranean environment.

### The user-friendly interface of the PASTORAL server

**Figure 5:**
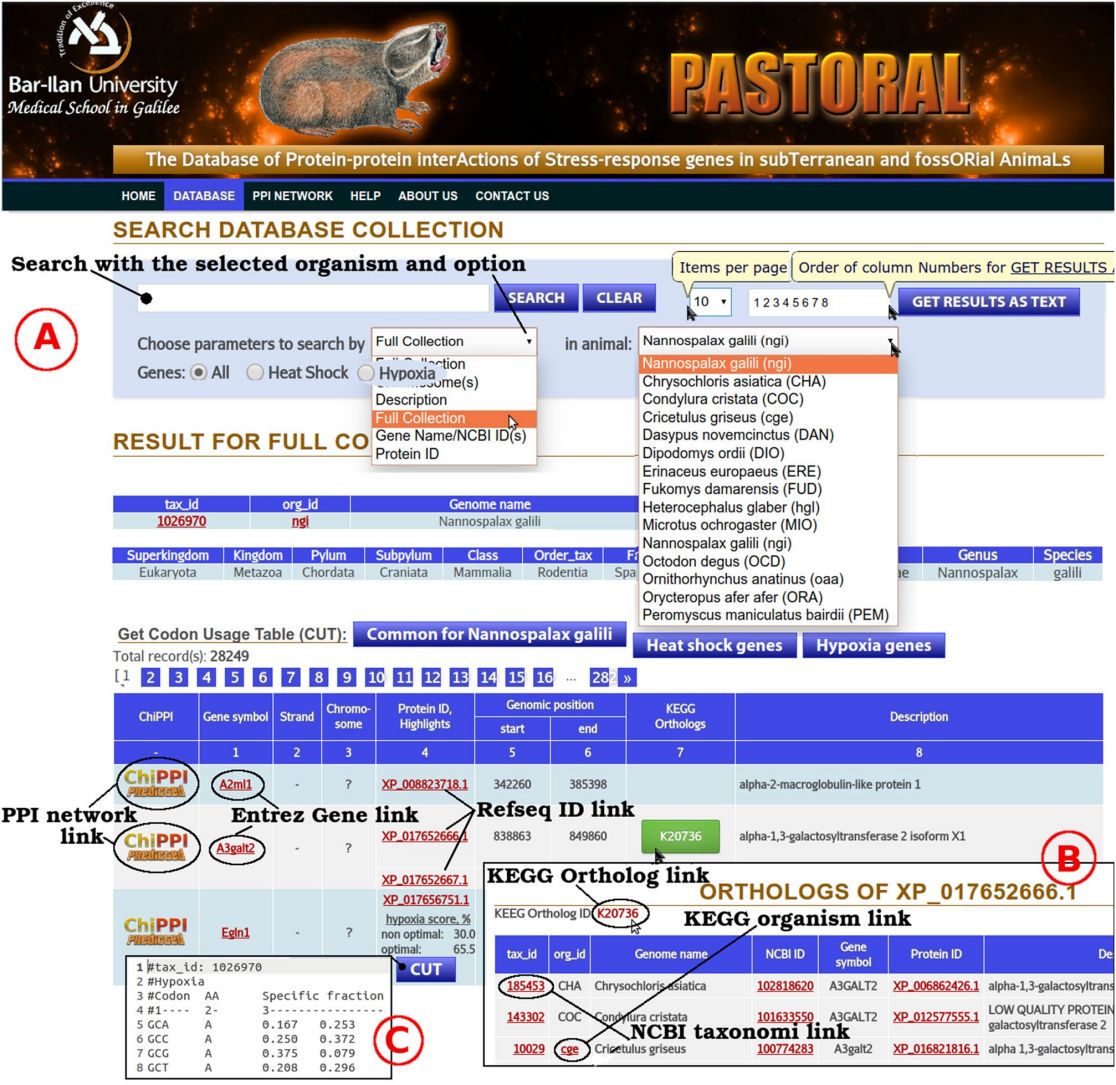
(**A**) The PASTORAL interface, showing querying and analysis features. (**B**) The pop-up window of protein orthologs. (**C**) An example of a partial codon usage table (CUT) for *Nannospalax galilli*. The codon usage table provides an overall %GC content and number of CDS from which the table is computed. AA, amino acid; Fraction, the proportion of usage of the codon among its degenerate set, i.e. the set of codons that code for an AA; Frequency, the expected number of codons, given the input sequence(s), per 1,000 bases; and Number, the raw number of occurrences of the codon in the input sequences.

Finally, we organized all our data in a dedicated resource, PASTORAL (Protein–Protein Interactions of Stress-Response Genes in Subterranean and Fossorial Animals). The PASTORAL database interface is user-friendly and accepts the following parameters for a selected animal as input query: Gene symbols, NCBI Entrez identifiers (NCBI_ID), protein ID, chromosomes, and gene descriptions. Upon an identified match of a search query, the user is directed to the entry webpage. From this page, all PPI data can be obtained (particularly for the heat-shock and hypoxia-related proteins) using annotations and the corresponding KEGG orthologs^17^ (see Fig. 5). Querying PASTORAL for two protein names (interactors) at most, or their NCBI_IDs, returns interactions for bi-level PPIs. Querying for three or more identifiers (maximum 380) returns interactions between these entities (single-level PPIs). The interactors can also be downloaded as a file in tab-delimited format. PASTORAL, written in mySQL, enables users to study proteins and their interactions in an intuitive workflow, as displayed in Fig. 5 and Figures S5–S7). Here, PASTORAL was used in an analysis involving NCBI_IDs for input proteins from 23 organisms listed in Table S1.

## Discussion and conclusions

The blind mole rat *Spalax galili* is an outstanding model for studying adaptation to life underground, with a remarkable predilection to disease including cancer^1,50^. A number of studies have shown that reduced mutations and chromosomal alterations are probably linked to hypoxia and hypercapnia, and that both may have significant roles in enhancing resistance to cancer in the blind mole rat^1^. With this in mind, the current study explored gene niche adaptation that results in the rewiring of PPI networks. We utilized fully sequenced genomes and proteomes of diverse taxa that inhabit similar ecological niches, namely, aboveground and underground habitats. These surroundings differ markedly in terms of environmental stresses. Accordingly, diverse organisms experience identical stresses imposed by virtue of their inhabiting a particular environment. We examined whether organisms sharing an ecological niche exhibit common attributes, distinct from those of organisms from a different ecological niche, specifically comparing subterranean and aboveground species.

We comprehensively assessed PPI network features between aboveground, fossorial, and subterranean organisms, considering various groups of orthologous proteins (KOs), and their domain content, namely, proteins with ordered, disordered, and mixed (ordered and disordered) regions. We evaluated PPI features, such as the total and average numbers of PPIs, as well as codon usage preferences by the encoding genes. We found that proteins with disordered regions generally form fewer PPIs and are encoded by genes that adopt more non-optimal codon usage, i.e., more negative CUPS than do counterpart proteins with ordered and mixed regions. Both PPIs and non-optimal codon usage were observed as more prevalent in subterranean than in fossorial and aboveground species. Taken together, these observations indicate that distantly related organisms inhabiting the same type of ecological niche is manifested in PPI networks and in the DNA and amino acid sequences of the interacting proteins. This is presumably a consequence of these organisms experiencing shared ecological stresses.

The above observations led us to hypothesize that substantial differences in the severity of stresses between above and underground habitats account for the great variance observed between organisms living in these habitats. This was reflected in differences between PPI networks and in the properties of interacting proteins. We presented evidence from PPIs of stress-related, hypoxia-related, heat-shock, and circadian proteins. All the organisms investigated demonstrated complete clustering according to PPI features, such that these clusters reflect the ecological niche-based classification of the organism considered. We confirmed that the distribution of hubs (key proteins) in ecology-specific PPI sub-networks accounts for such clustering by ecology. Accordingly, the key proteins (hubs) and essential interactions in the PPI networks of PAS (Per-Arnt-Sim^51^) domain -containing proteins are central players in environmental stress response pathways, such as hypoxia, heat-shock, and circadian and dioxin response pathways. This demonstrated the applicability of PPI network analyze is to understanding biological phenomena. Together, our results allude to the intimate relation between ecology and evolution, in general, and convergent evolution, in particular, due to the shared stress experienced by species confined to the same ecology. Finally, we organized all PPIs and codon usage data in a dedicated user-friendly resource, PASTORAL, which provides evolutionary biologists an extensive and comprehensive tool to study convergent evolution related to stress responses and other essential cellular processes.

## Materials and methods

### Data resources

Five core resources were used: Entrez/NCBI^52^, KEGG^17^, BioGrid (release 3.4.163)^16^, Pfam (release 31.0)^53^, and the Gene Ontology^54,55^ (GO) consortium. Complete genomes, proteomes, and coding sequences were obtained from NCBI. Ortholog annotations were obtained from KEGG, whereas annotations for organisms not included in the KEGG database, coded by an upper-case three-letter code, were obtained using the BLASTKOALA web-tool^17,56^. Annotations from KEGG included ortholog (KO) groups (https://rest.kegg.jp/list/ko) and GO annotations (linkDB within KEGG). Annotations for Pfam domains were retrieved from Pfam (release 31.0)^53^. We collected data for the following 23 organisms: ten fossorial species—*Cricetulus griseus* (Chinese hamster, cge), *Condylura cristata* (star-nosed mole, COC)^57^, *Dasypus novemcinctus* (nine-banded armadillo, DAN), *Microtus ochrogaster* (prairie vole, MIO), *Octodon degus* (common degu, OCD), *Peromyscus maniculatus bairdii* (deer mouse, PEM), *Dipodomys ordii* (Ord's kangaroo rat, DIO), *Camponotus floridanus* (Florida carpenter ant, cfo), *Manis javanica* (Malayan pangolin, mjv), and *Solenopsis invicta* (red fire ant, soc); nine subterranean species—*Chrysochloris asiatica* (Cape golden mole, CHA), *Fukomys damarensis* (Damara mole rat, FUD), *Heterocephalus glaber* (naked mole-rat ,hgl), *Nannospalax galili* (blind mole rat, ngi), *Astyanax mexicanus* (Mexican tetra, ASM), *Cryptotermes secundus* (drywood termite, CRS), *Zootermopsis nevadensis* (dampwood termite, zne), and *Myotis lucifugus* (little brown bat, MYL); and 13 organisms that live aboveground—*Erinaceus europaeus* (European hedgehog, ERE), *Ornithorhynchus anatinus* (platypus, oaa), *Orycteropus afer* (aardvark, ORA), *Homo sapiens* (human, hsa), *Mus musculus* (mouse, mmu), *Rattus norvegicus* (rat, rno), *Pan troglodytes* (chimpanzee, ptr), *Gallus gallus* (chicken, gga*), Felis catus* (cat, fca), *Drosophila melanogaster* (fruitfly, dme), *Bos taurus* (cow, bta), *Sus scrofa* (swine, ssc), and *Danio rerio* (zebrafish, dre).

### Conservation and point mutations in protein domain sequences

Protein domains, delineated by coordinates identified by Perl scripts written in the lab, along with the Pfam search tool^53^, were extracted using the *extractseq* program (EMBOSS:6.6.0.0) with the ‘regions’ option. The highest scoring domain was reserved for multiple sequence alignment analysis, thus ensuring a single sequence per organism. Only domains conserved among 10 or more animals were analyzed. Multiple sequence alignment was performed using T-Coffee^58^ with default parameters. Statistical analysis was performed using R, and hierarchical clustering was performed and assessed using pvclust^59^.

### PPIs of stress response genes

We identified PPIs encoded by all stress-response genes using the ChiPPI tool, which we previously described^15^. ChiPPI assumes that PPIs can be approximated by calculating the propensity of discreet domains by means of a pre-computed domain-domain co-occurrence table (DDCOT) from all interactions in BioGrid^16^. A new PPI network was generated based on the DDCOT for each organism examined in this study. Thus, we identified domain-domain co-occurrences for each PPI to detect potential interacting proteins that reflect the overall structure of the PPI network^15^. Additionally, we used the agglomerative hierarchical clustering method to classify the stress-response genes for all 32 organisms in PASTORAL.

### Domain prediction method

The Pfam database represents protein domains as profile-hidden Markov models (HMM)^53^. Accordingly, protein sequences were searched by HMMER (version 3.2.1, 13 June 2018)^60^ with Pfam-provided HMM profiles, to predict protein domains. In addition, disordered regions were predicted based on the redox state using IUPred2A^44^ Predicted disordered regions are treated as a single generic type (DISORDERED) for the generation of PPI networks. Hence, disordered regions are incorporated within a PPI model as an additional DISORDERED domain, a component of domain-domain co-occurrence scores from which PPIs are predicted.

### Codon usage preference score (CUPS)

Previously, we found that genes adopt non-optimal codon usage to modulate protein expression in a cell-cycle dependent manner^25^. The distinction between optimal and non-optimal codons refers to the strength of the codon-anti-codon interaction, where optimal and non-optimal codons have high and low affinity, respectively, due to the “wobble” effect^24,25^. To quantify codon usage preferences, we devised optimal and non-optimal codon usage scores for the respective optimal and non-optimal codons. Moreover, we defined the difference between optimal and non-optimal as a tendency score. A positive tendency score represents an optimal codon usage preference, while negative values represent non-optimal codon preferences. Both optimal and non-optimal codon usage scores are computed from codon frequencies obtained from a Codon Usage Table (CUT), calculated using the cusp program (EMBOSS:6.6.0.0) for a set, of coding sequence (CDSs).

1 $$Optimal\left(S\right)=100\cdot {\left[1+\frac{\left|{Cod}_{Opt}\right|\cdot {\sum }_{j\in {Cod}_{nonOpt}}{\nu \left(S\right)}_{j}}{\left|{Cod}_{nonOpt}\right|\cdot {\sum }_{i\in {Cod}_{Opt}}{\nu \left(S\right)}_{i}}\right]}^{-1}$$

2 $$nonOptimal\left(S\right)=100\cdot {\left[1+\frac{\left|{Cod}_{nonOpt}\right|\cdot {\sum }_{i\in {Cod}_{Opt}}{\nu \left(S\right)}_{i}}{\left|{Cod}_{Opt}\right|\cdot {\sum }_{j\in {Cod}_{nonOpt}}{\nu \left(S\right)}_{j}}\right]}^{-1}$$

Here ν is the frequency of codons obtained from the Codon Usage Table (CUT), *Cod*_*Opt*_ and *Cod*_*nonOpt*_ are sets of optimal and non-optimal codons^25^, respectively, |*Cod*_*Opt*_| and |*Cod*_*nonOpt*_| are the numbers of elements of these sets and S is a set of CDSs.

3 $$tendency\left(S\right)=Optimal\left(S\right)-nonOptimal\left(S\right)$$

where tendency is referred to as a codon usage preference score (CUPS).

To evaluate the significance of codon usage (CUPS) among subterranean and fossorial organisms, relative to aboveground species (observed values), bootstrap analysis was performed. Bootstrap analysis consisted of generating 10,00 random groups of 516 KOs (bootstrap replicates) and calculating p-values associated with observed values.

Codon usage was calculated as the summed areas under density distribution, i.e., negative (non-optimal) and positive (optimal) CUPS. These areas were scaled according to aboveground animals. Bootstrap replicates were generated by assigning a random number to each KO, using the random_normal() function of the Math::Random Perl module, and selecting the first 516 KO from a sorted list.

P-values were calculated as a proportion of bootstrap replicates, with codon usage equal or exceeding observed codon usage. *p* values of 1% were considered significant.

## Supplementary information

Supplementary information

Supplementary Table S1

Supplementary Table S2

Supplementary Table S3

Supplementary Table S4

Supplementary Table S5

Supplementary Table S6

Supplementary Table S7

Supplementary Table S8

Supplementary Table S11

Supplementary Table S12

Supplementary Table S13
