# Supplementary material for: Design principles of gene evolution for niche adaptation through changes in protein–protein interaction networks
Source: Sci Rep. 2020 Sep 24;10:15628. doi: 10.1038/s41598-020-71976-x (PMC7519090; doi:10.1038/s41598-020-71976-x)
Supplement: Supplementary file 1 — Supplementary information [file 41598_2020_71976_MOESM1_ESM.pdf]

Supplementary Material

Design principles of gene evolution for niche adaptation through changes in protein-protein interaction networks

Gon Carmi[1,†], Somnath Tagore[1,2,†], Alessandro Gorohovski[1,†], Aviad Sivan[1], Dorith Raviv-Shay[1], Milana Frenkel-Morgenstern[1*]

[1] The Azrieli Faculty of Medicine, Bar-Ilan University, 8 Henrietta Szold St, Safed 13195, ISRAEL
[2] Current address: Department of Systems Biology, Columbia University Medical Center, Herbert Irving Cancer Research Center, New York, USA
* Correspondence: milana.morgenstern@biu.ac.il
† These authors contributed equally to this work.

Supplementary Results

Species data collection

We collected, calculated, and organized various data for all organisms in the PASTORAL data resource, including general information and PPI network statistics (Table S1). General information includes ecology (fossorial (F), subterranean (S), and aboveground (A)), organism name, organism NCBI-ID, and KEGG KO. Moreover, the number of genes in total, HomoloGene (NCBI database) and the number of KO groups (total, unique) are included in PASTORAL. The PPI statistics include the number of orthologous proteins (KEGG or HomoloGene ID) that appear in PPI, protein domains and the number of interactors from BioGrid [16] and PPIs predicted by ChiPPI[15]. The clustering coefficient of the largest linked PPI network for organisms and the average number of interactors per protein were calculated. In addition, ecology level averages were included (Table S1). The total number of interactors (Table S1) should be the sum of the number of interactors in ordered, mixed, and disordered sets. However, differences arise because some data did not have sequences to predict protein structure. For example, in dme (fly), gga (chicken), hsa (human), mmu (mouse), and rno (rat), some proteins have non-protein interactors (Table S2). A full analysis of human PPIs in the BioGrid database revealed that 25,206 (~8.86%) of PPIs (pairs of interactors) lack domains for one of the interactors. These interactors are 949 non-coding genes that lack any associated protein-coding sequences. Therefore, a disordered region search was not performed on these gene products due to the absence of amino acid sequences (Table S3).

Ecology-level estimates of PPI features were common to all organisms

In the current study, we hypothesized that fully orthologous proteins, namely KO groups from the KEGG database [17] that are common to all organisms, would have similar PPI

networks with similar ecology level estimates of PPI network properties, for example, global/individual clustering coefficients. To test this, we collected 516 KOs common to all organisms and computed PPI networks for orthologous proteins that are common to all organisms. We then calculated associated PPI properties, namely, the number of interactors and the number of PPIs, as well as global/individual clustering coefficients. We found similar values among ecologies when they were considered as a whole (Figure S1, Figure S2). What we saw is in a group is that A>FS, namely, KO groups for which clustering coefficients are greater show significant decrease in S relative to A and the mirror image in (F|S)>A shows a significant increase in S relative to A.

In particular, among aggregated values, differences between ecologies in the PPI properties examined were non-significant (p-value >0.05, Wilcoxon rank sum test with continuity correction; Table S4, Figure S1). We found that PPI network properties of individual orthologous proteins varied among ecologies, namely, aboveground (A), fossorial (F), and subterranean (S). For example, individual clustering coefficients of CTP Synthase 2 (CTPS2 protein) were ‘A' (0.626), 'F' (0.598), and 'S' (0.580), i.e. ‘A>F>S’. Therefore, we portioned proteins into groups based on values of cluster coefficients within each ecology, e.g., 'A>FS' (A>F and A>S); '(F|S)>A' (i.e., 'FS>A' (F>A and S>A); 'S>A>F'; and 'F>A>S').

Testing our hypothesis on a subset of these orthologous proteins, we observed for the A>FS group, relative to aboveground animals, 30.55% and 16.08% decreased individual and global clustering coefficients among subterranean animals, respectively (p-value < 0.0012, Wilcoxon rank sum test with continuity correction; Table S4). For ‘(F|S)>A’, relative to aboveground animals, 23.48% % and 18.4% increased individual and global clustering coefficients among subterranean animals, respectively (p-value < 0.001, Wilcoxon rank sum test with continuity correction; Table S4). These differences were derived from selecting subsets of orthologous proteins. Moreover, the significant differences observed were only apparent in clustering coefficients (individual and global), and not in the number of interactors. Moreover, the interactions (PPIs) were due to the computation of cluster coefficients, which included second level interactions that involved orthologous proteins not common to all organisms (Table S4). These results indicate greater differences in individual/global clustering coefficients between aboveground and subterranean animals, among subsets of orthologous proteins, namely, ‘A>FS’.

Hub distribution discriminates between PPIs of different ecologies

In PPI networks, hubs are nodes that impair network structural integrity when removed, such as by fragmentation of the network into sub-networks. These hubs are defined by a network

topological measure called "relative connectivity" [61]. Hubs are the major contributors to the overall shape of PPI networks, and can be classified into two types, 'Party' and 'Date'. Party hubs represent group proteins that perform single functions, while 'Date' hubs connect and coordinate between groups that perform diverse functions [14,15,62-65].

We hypothesized that PPI sub-networks would cluster by ecology. To study adaptation, we focused on a hypoxia-related protein (hypoxia-inducible factor-2alpha, HIF2A), also known as endothelial PAS domain-containing protein 1 (EPAS1), which belongs to KO group 09095. This protein family is common to all 32 organisms examined, except for *Drosophila melanogaster* (fruit fly). PAS is an acronym of the three proteins, Per-Arnt-Sim [66-75], originally referred to as the PAS domain. This domain is conserved among bacteria, archaea, and eukaryotes, and is involved in the hypoxia, circadian, and dioxin pathways [51]. PAS can bind small molecules, such as oxygen-carrying heme [76,77] and mediate PPIs [51]. For the EPAS1 protein family, we calculated an identity matrix (point mutation), derived from multiple sequence alignment analysis [58] performed on PAS domain sequences, and also studied hubs in the networks.

To examine whether PPI sub-network hubs cluster by ecology, we first built PPI networks for the EPAS1 protein family in 31 of the studied organisms (Figure 3, Figure S3). The network size, i.e., number of interactors, was large in aboveground animals (range: 95-99), smaller in fossorial animals (range: 80-93), and smallest in subterranean animals (range: 45-64). The network clustering coefficients were 0.58 (aboveground), 0.62 (fossorial), and 0.74 (subterranean). The subterranean organisms had 1.28 times more condenser networks than the organisms from other habitats (p-value = 0.025, Pearson's $\chi 2$-test).

To understand the condensing property of the subterranean PPI network, we examined the central nodes in the networks or hubs that differ among ecologies. To this end, we used the clustering coefficient as a parameter to distinguish Date and Party hubs, wherein Party hubs tend to have a higher CC as they act in an intra-modular manner. We detected, in total, nine hubs in the EPAS1 networks (Figure S3), six of which are 'Date' hubs: EPAS1, HIF1A, EP300, SMAD3, XPO1, and EWSR1, and three of which are 'Party' hubs: MED4, MED23, and CREBBP (Figure S3). We observed that EPAS1 and HIF1A, which are key proteins in the hypoxia pathway [51], are common Date hubs in all ecologies (marked red, Figure S3). However, we found three Date hubs (SMAD3, XPO1, and EWSR1), and one Party hub (CREBBP) that are unique to subterranean animals (Figure S3). EPAS1 and HIF1A form a dimer as part of the hypoxia pathway [51]. This is consistent with these proteins being Date hubs. Moreover, EPAS1 was identified as having a strong selection signature within the

highest altitude population of the subterranean Zokor, *Myospalax baileyi,* compared with two such populations that habituate lower altitudes [78]. This indicates that our results are applicable to other subterranean animals. Furthermore, protein domain PPI network methodology suggests a plausible mechanism at the protein domain level, rather than at the amino acid level of point mutations. Thus, the distribution of Date and Party hubs, both in numbers and types, discriminates between PPIs of different ecologies. Accordingly, our results confirm our hypothesis that PPI sub-networks cluster by ecology. We speculate on a particular link for the "shuffling" of protein domains with global changes in PPI networks. This could be mediated by changes in the distribution of hubs that are ecologically specific, as a response to underground stresses (Figure S3).

Proteins with disordered regions adopt non-optimal codon usage and form fewer PPIs

We further evaluated aggregates of PPIs and measures of codon usage preferences (CUPS) across ecologies and protein regions (ordered, disordered, and mixed). Specifically, within each ecology, we compared (i) aggregates between proteins with ordered, disordered, and mixed regions and (ii) aggregates between ecologies. Both analyses (i) and (ii), were performed on all proteins (PPIs, Table S2; CUPS,Table S8). In general, within each ecology, proteins with ordered and mixed regions formed more PPIs, on average, than proteins with disordered regions, e.g., the average number of PPIs for proteins of subterranean animals with ordered, disordered, and mixed regions were 26.32, 16.95, and 35.80, respectively, that is 35.6% less PPIs from disordered than ordered regions (p-value < 2.2e-16, Wilcoxon rank sum test with continuity correction; Table S2). Additionally, proteins containing ordered, disordered, and mixed regions in subterranean animals showed average CUPS of 16.25, 15.96, and 17.28, respectively (p-value = 5.086e-07 for ordered vs. mixed; p-value = 0.02338 for mixed vs. disordered, Wilcoxon rank sum test with continuity correction; Table S8). Similar results were observed for proteins from other ecologies and are indicated in Table S9 (non-significant at p-value >0.05, Wilcoxon rank sum test with continuity correction, derived from Table S2 and Table S8 for PPI and CUPS data, respectively). Moreover, proteins with ordered and mixed regions showed more interactors per protein, on average, than proteins with disordered regions (fold change (mixed/disordered) = 15.02, fold change (ordered/disordered) = 5.96; Table S2). Proteins of subterranean animals with any region type were found to adopt fewer optimal codon usage preferences (Table S9), for example, CUPS observed for proteins with disordered regions were 18.65 (fossorial), 15.96 (subterranean), and 20.96 (aboveground) (p-value = 0.0005078, fossorial vs subterranean; p-value = 8.758e-05, fossorial vs. aboveground; and p-value = 9.507e-14, subterranean vs aboveground, Wilcoxon rank sum test with continuity

correction; Table S8). However, for proteins with disordered regions, the number of PPIs did not differ significantly between ecologies (Table S2, Table S9).

FIGURES

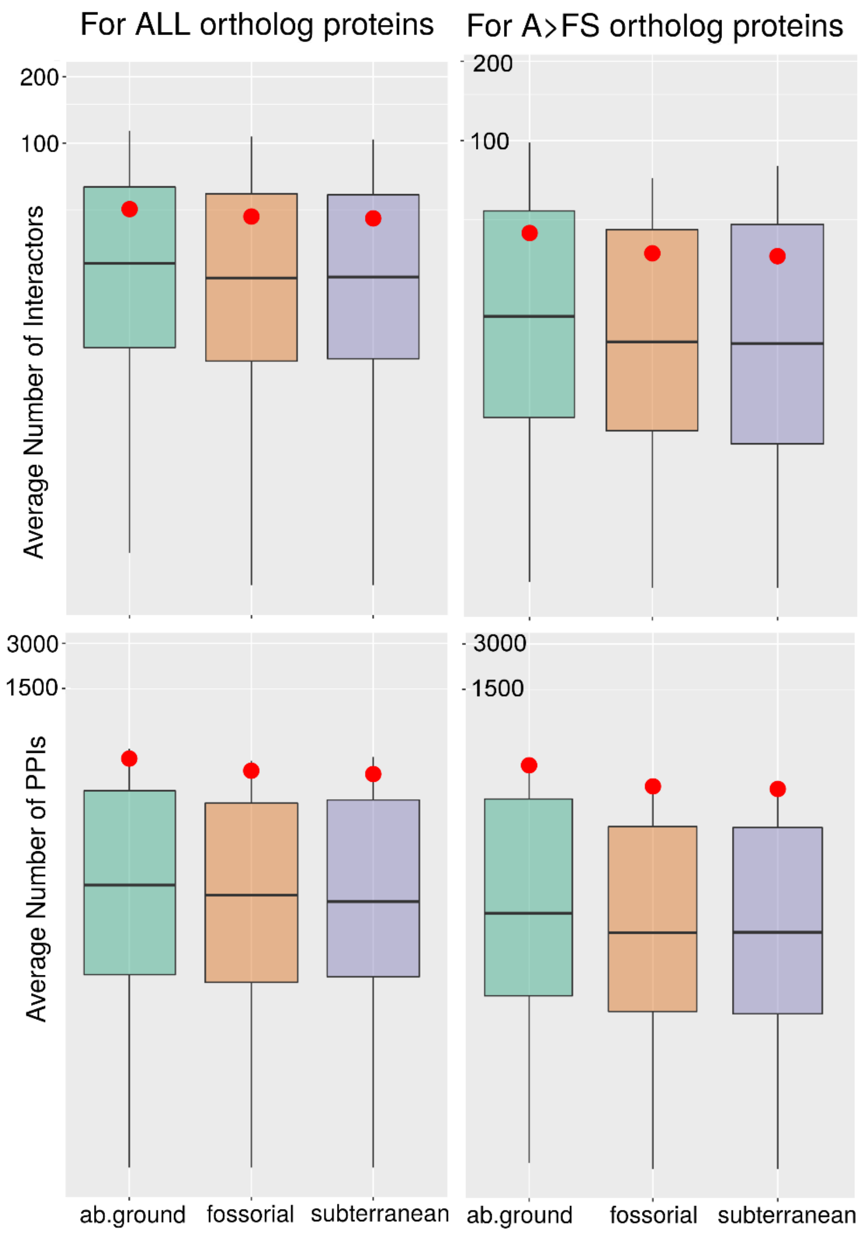

Figure S1: Comparison of protein-protein interaction (PPI) network features (y-axis, log scale) among ecologies (x-axis), comprising two groups of orthologous proteins, full set (all) and A>FS, left and right panels, respectively. A>FS are proteins for which the values of cluster coefficients satisfying the relation among ecologies were ab.ground (aboveground A), fossorial (F), and subterranean (S). PPI represents the mean number of interactors/interactions (PPIs). The red dots are the mean values. All orthologous proteins are common to all organisms.

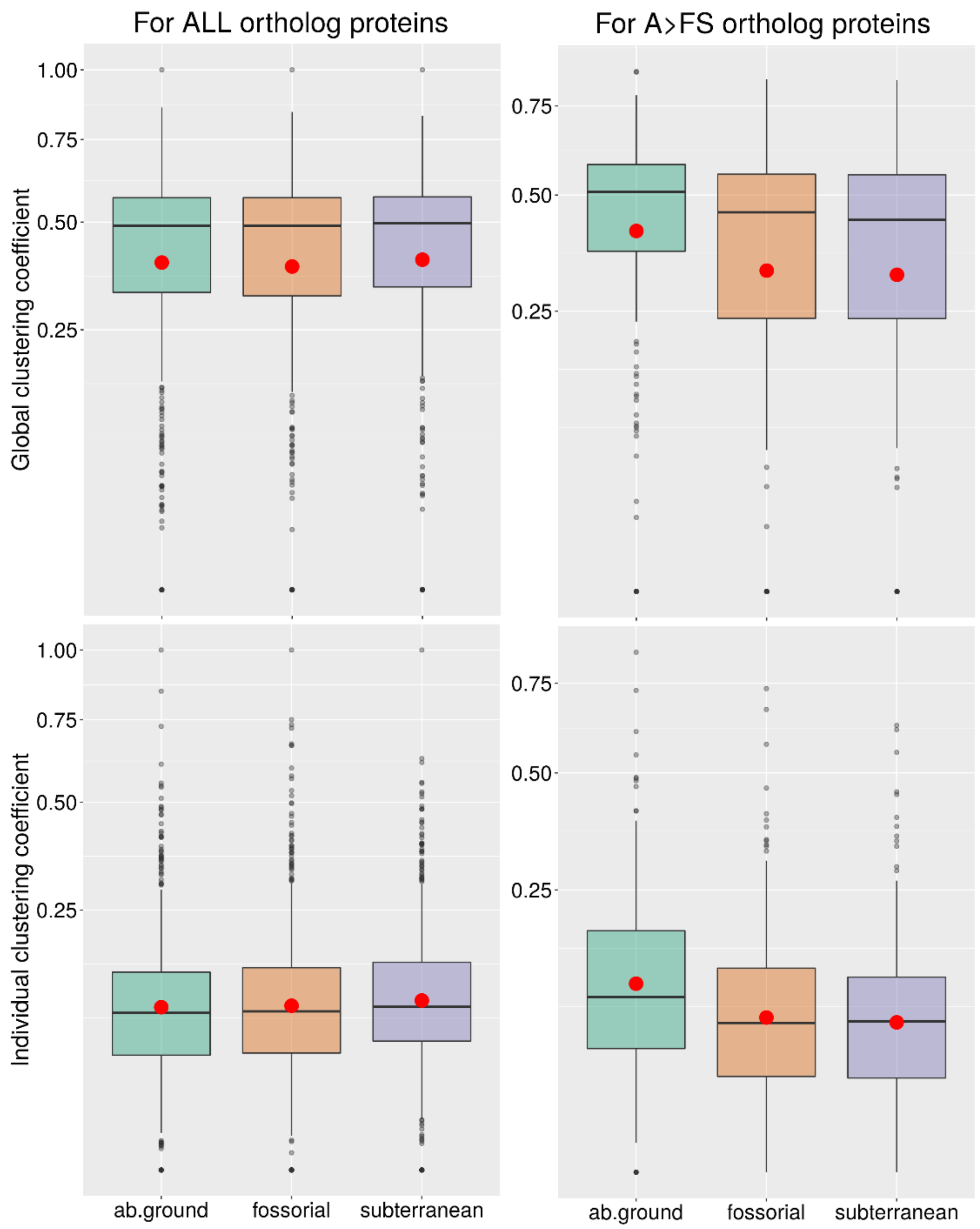

Figure S2: Comparison of protein-protein interaction (PPI) network features (y-axis, square root scale) among ecologies (x-axis), comprising two groups of ortholog proteins, full set (all) and A>FS, left and right panels, respectively. A>FS are proteins for which the values of cluster coefficients satisfying the relation among ecologies were ab.ground (aboveground A), fossorial (F), and subterranean (S). PPI features are global / individual clustering coefficient. The red dot is the mean value. All orthologous proteins are common to all organisms.

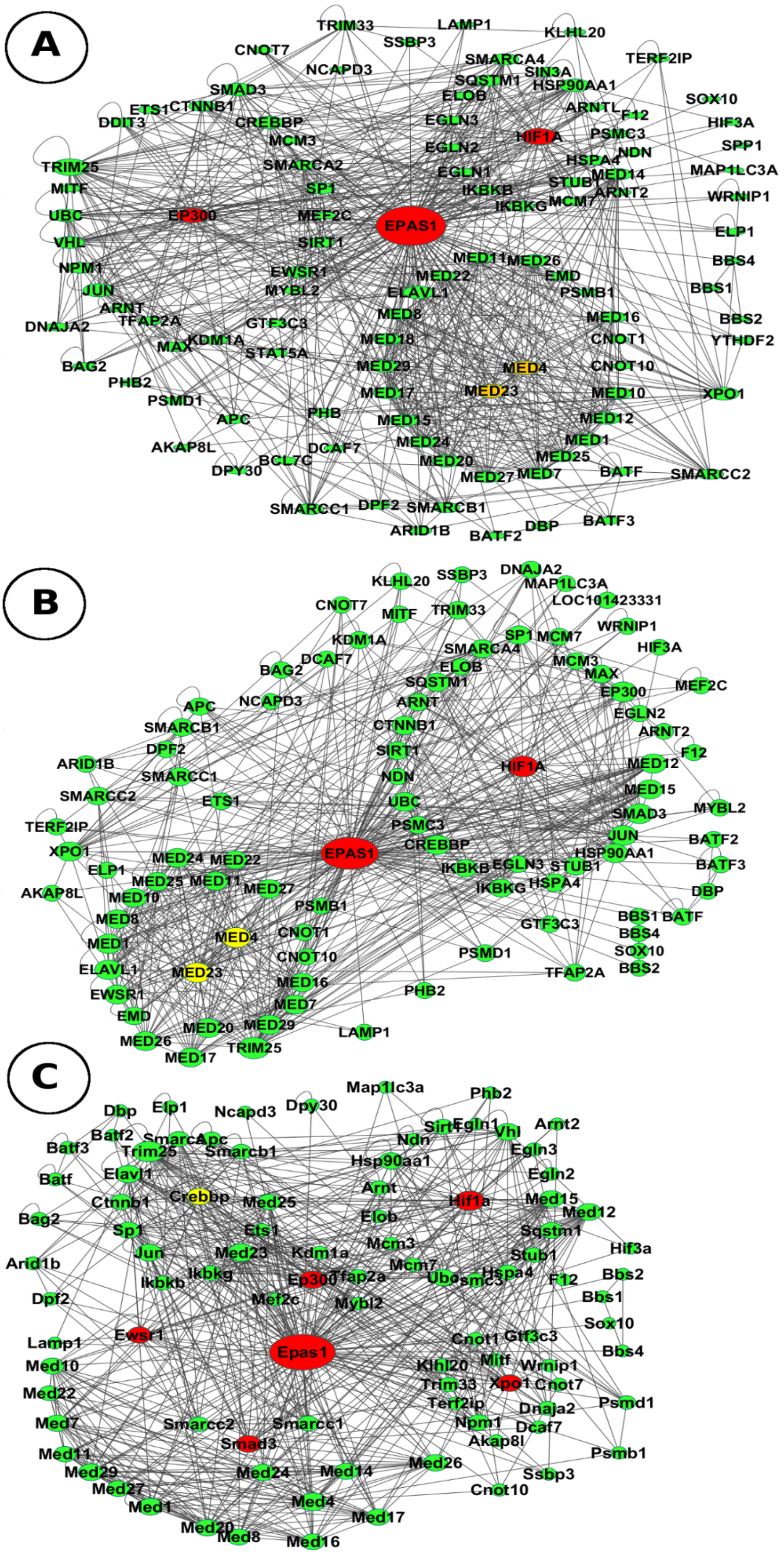
A
TRIM33
LAMP1
KLHL20
CNOT7
SSBP3
SMARCA4
TERF2IP
NCAPD3
SIN3A
SQSTM1
HSP90AA1
SMAD3
ELOB
SOX10
ETS1
CTNNB1
ARNTL
F12
DDIT3
CREBBP
EGLN3
HIF3A
HIF1A
PSMC3
MCM3
EGLN2
NDN
SPP1
TRIM25
SMARCA2
HSPA4
EGLN1
MED14
MAP1LC3A
MITF
SP1
IKBKB
STUB1
ARNT2
UBC
EP300
MEF2C
IKBKG
MCM7
WRNIP1
EPAS1
VHL
SIRT1
ELP1
MED11
MED26
BBS4
NPM1
EWSR1
MED22
EMD
JUN
MYBL2
ELAVL1
PSMB1
BBS1
ARNT
MED8
MED16
BBS2
DNAJA2
TFAP2A
GTF3C3
KDM1A
MED18
CNOT1
YTHDF2
MAX
STAT5A
BAG2
MED29
MED4
CNOT10
PHB2
MED17
MED23
MED10
XPO1
PSMD1
APC
PHB
MED15
MED12
AKAP8L
DCAF7
MED24
MED1
BCL7C
MED25
DPY30
MED20
MED27
MED7
BATF
SMARCC2
SMARCC1
DPF2
SMARCB1
ARID1B
BATF3
DBP
BATF2
B
DNAJA2
KLHL20
SSBP3
MAP1LC3A
LOC101423331
CNOT7
MITF
TRIM33
WRNIP1
KDM1A
SP1
MCM7
HIF3A
SMARCA4
DCAF7
BAG2
ELOB
MCM3
MAX
SQSTM1
MEF2C
NCAPD3
EP300
APC
ARNT
EGLN2
SMARCB1
CTNNB1
ARNT2
ARID1B
DPF2
SIRT1
HIF1A
F12
MED12
SMARCC1
NDN
MED15
SMARCC2
ETS1
UBC
SMAD3
MYBL2
TERF2IP
PSMC3
JUN
BATF2
XPO1
MED24
MED22
EPAS1
CREBBP
HSP90AA1
ELP1
IKBKB
EGLN3
STUB1
BATF3
MED25
MED11
MED27
AKAP8L
MED10
IKBKG
HSPA4
DBP
PSMB1
MED8
BBS1
BATF
GTF3C3
MED1
MED4
CNOT1
BBS4
SOX10
ELAVL1
CNOT10
PSMD1
MED23
BBS2
TFAP2A
MED16
EWSR1
PHB2
MED7
EMD
MED29
MED20
LAMP1
MED26
TRIM25
MED17
C
Map1lc3a
Phb2
Dbp
Elp1
Ncapd3
Dpy30
Sirt1
Egln1
Arnt2
Batf2
Apc
Vhl
Ndn
Batf3
Trim25
Smarcb1
Egln3
Hsp90aa1
Batf
Elavl1
Egln2
Arnt
Crebbp
Med25
Hif1a
Med15
Ctnnb1
Med12
Bag2
Elob
Sp1
Ets1
Sqstm1
Mcm3
Hif3a
Arid1b
Jun
Med23
Kdm1a
Stub1
Ikbkg
Mcm7
Ikbkb
Ep300
Tfap2a
Ubc
Hspa4
F12
Bbs2
Dpf2
Mef2c
Mybl2
Bbs1
Ewsr1
Sox10
Lamp1
Cnot1
Gtf3c3
Bbs4
Med10
Epas1
Klhl20
Mitf
Wrnip1
Trim33
Xpo1
Cnot7
Med22
Terf2ip
Dnaja2
Psmd1
Med7
Smarcc2
Smarcc1
Npm1
Dcaf7
Smad3
Akap8l
Med11
Psmb1
Med26
Med29
Med24
Med14
Ssbp3
Med27
Cnot10
Med1
Med4
Med17
Med20
Med8
Med16

Figure S3 Hub analysis on PPI networks of EPAS1 from A) aboveground (human), B) fossorial (*Cricetulus griseus* (Chinese hamster)) and C) subterranean (*Nannospalax galili* (blind mole rat)) species. 'Date' (n=6) and 'Party (n=3) hubs are indicated in red and yellow, respectively. Party hubs represent group proteins and perform single functions, while 'Date hubs connect and coordinate between such groups and perform diverse functions [14,15,62-65]. Interestingly, we observed three Date hubs (SMAD3, XPO1, and EWSR1) and one Party hub (CREBBP) unique to subterranean animals. Moreover, MED4 and MED3 were not detected as Party hubs in subterranean animals. The distribution of Date and Party hubs, both in numbers and type, discriminates between PPIs of different ecologies.

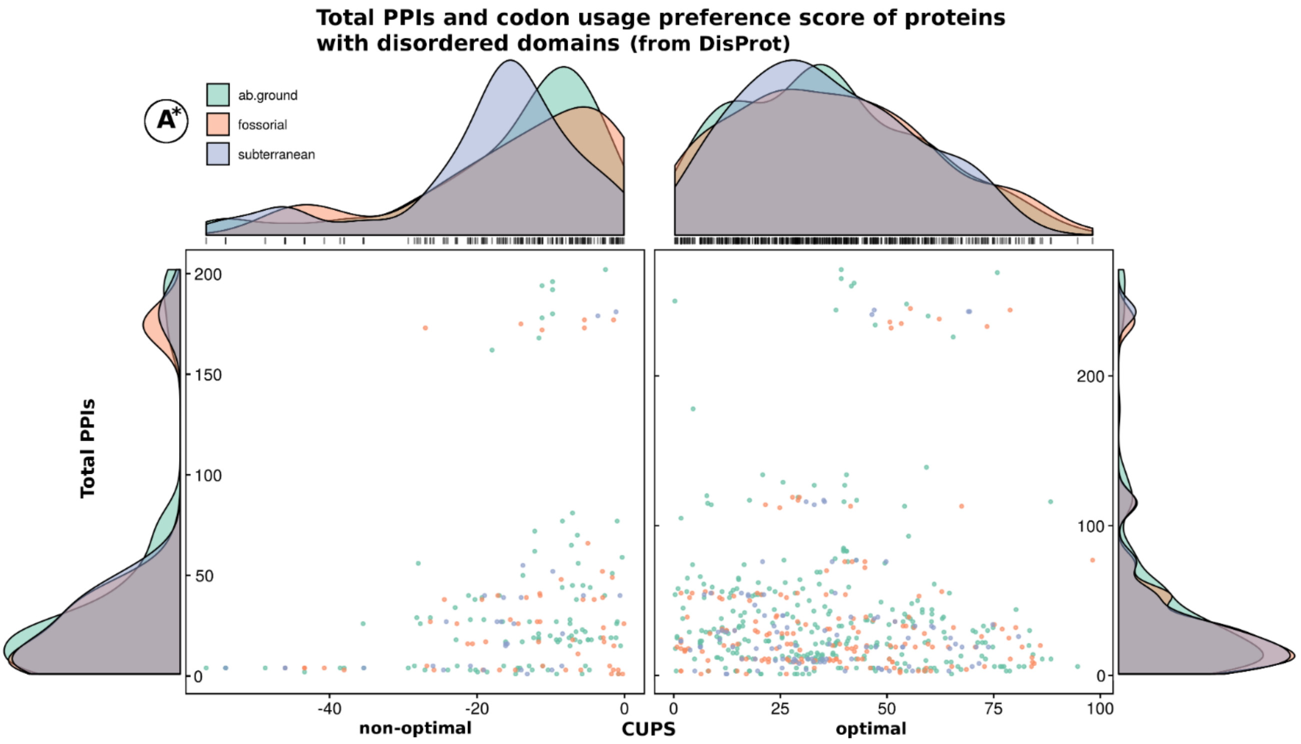

Figure S4: Total PPIs and codon usage preference score with density plots for protein with disordered domains (from DisProt) stratified by ecology (aboveground, lime green (#66c2a5)*, fossorial, soft orange (#fc8d62)* and subterranean, light blue (#8da0cb)*. Subterranean animals adopt extreme non-optimal codon usage preferences and form less PPIs, relative to aboveground and fossorial ecologies. *Hexadecimal color number.

STATISTICS AND NETWORK STRUCTURE INFORMATION

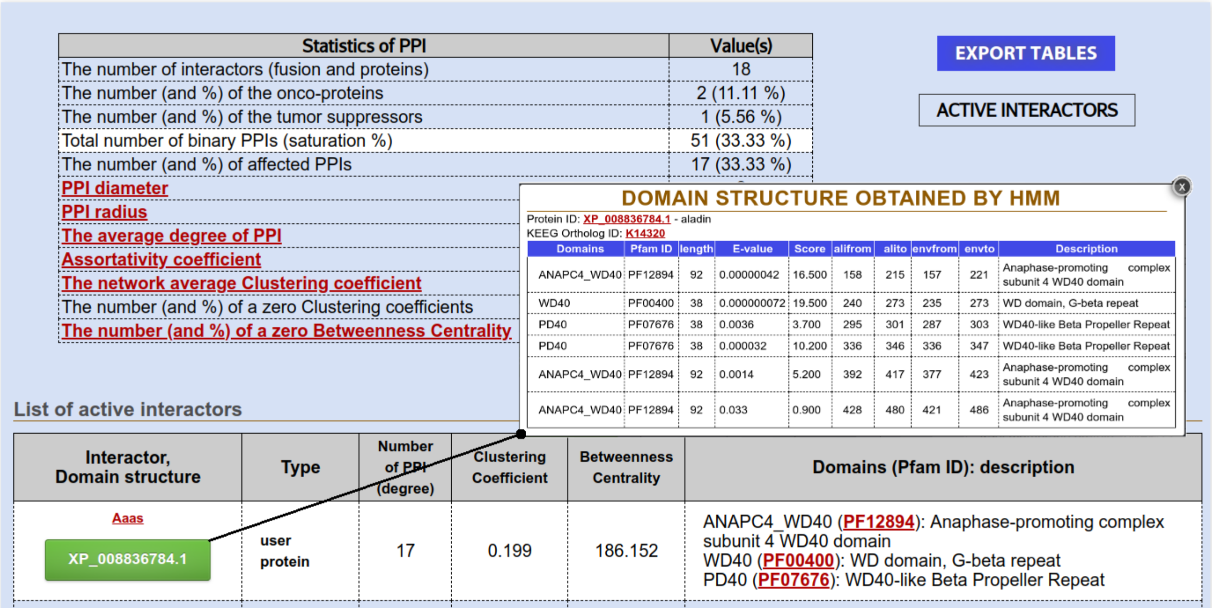

Statistics of PPI	Value(s)
The number of interactors (fusion and proteins)	18
The number (and %) of the onco-proteins	2 (11.11 %)
The number (and %) of the tumor suppressors	1 (5.56 %)
Total number of binary PPIs (saturation %)	51 (33.33 %)
The number (and %) of affected PPIs	17 (33.33 %)
PPI diameter	
PPI radius	
The average degree of PPI	
Assortativity coefficient	
The network average Clustering coefficient	
The number (and %) of a zero Clustering coefficients	
The number (and %) of a zero Betweenness Centrality	

EXPORT TABLES

ACTIVE INTERACTORS

DOMAIN STRUCTURE OBTAINED BY HMM

Protein ID: XP_008836784.1 - aladin

KEEG Ortholog ID: K14320

Domains	Pfam ID	length	E-value	Score	alifrom	alito	envfrom	envto	Description
ANAPC4_WD40	PF12894	92	0.00000042	16.500	158	215	157	221	Anaphase-promoting complex subunit 4 WD40 domain
WD40	PF00400	38	0.000000072	19.500	240	273	235	273	WD domain, G-beta repeat
PD40	PF07676	38	0.0036	3.700	295	301	287	303	WD40-like Beta Propeller Repeat
PD40	PF07676	38	0.000032	10.200	336	346	336	347	WD40-like Beta Propeller Repeat
ANAPC4_WD40	PF12894	92	0.0014	5.200	392	417	377	423	Anaphase-promoting complex subunit 4 WD40 domain
ANAPC4_WD40	PF12894	92	0.033	0.900	428	480	421	486	Anaphase-promoting complex subunit 4 WD40 domain

List of active interactors

Interactor, Domain structure	Type	Number of PPI (degree)	Clustering Coefficient	Betweenness Centrality	Domains (Pfam ID): description
Aaas XP_008836784.1	user protein	17	0.199	186.152	ANAPC4_WD40 (PF12894): Anaphase-promoting complex subunit 4 WD40 domain<br>WD40 (PF00400): WD domain, G-beta repeat<br>PD40 (PF07676): WD40-like Beta Propeller Repeat

Figure S1: PPI network statistics (top panel) and structure information about active interactors. Lower panel provides information about active interactors.

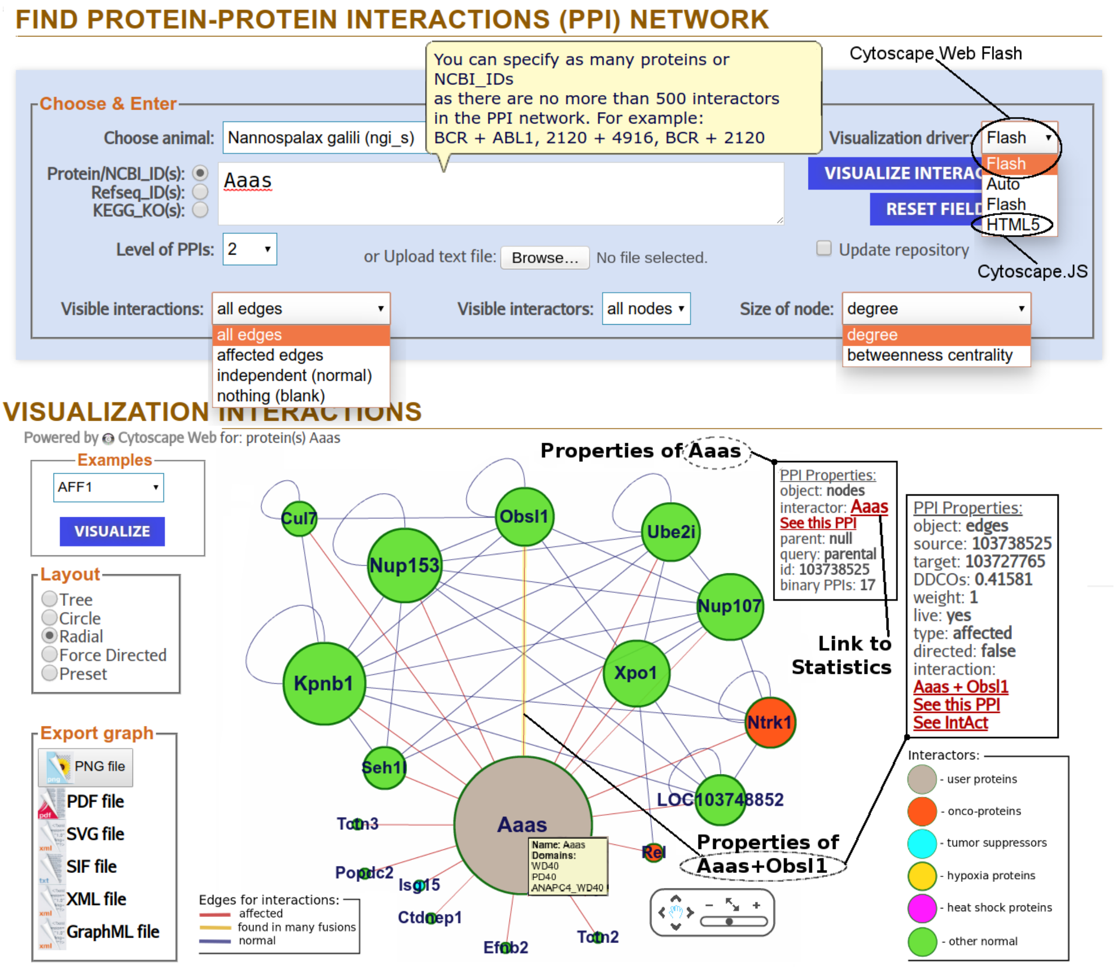

Figure S2: The interface of PPI Network with querying and analysis features. PPI networks can be searched using either protein names or NCBI_IDs. The interactions can be visualized in several layouts: Breadth first, Circle, Concentric, Case, Force Directed, Grid, Radial, Random, and Tree. Various network properties can be studied, such as the number of interacting pairs, affected interactors, and diameter, clustering coefficient, and centrality measures. Finally, for each interaction, cross-links are present for databases like IntAct, Pfam, and NCBI. The statistical components and the network structure information also appear on the PASTORAL website at the bottom (Figure S1).

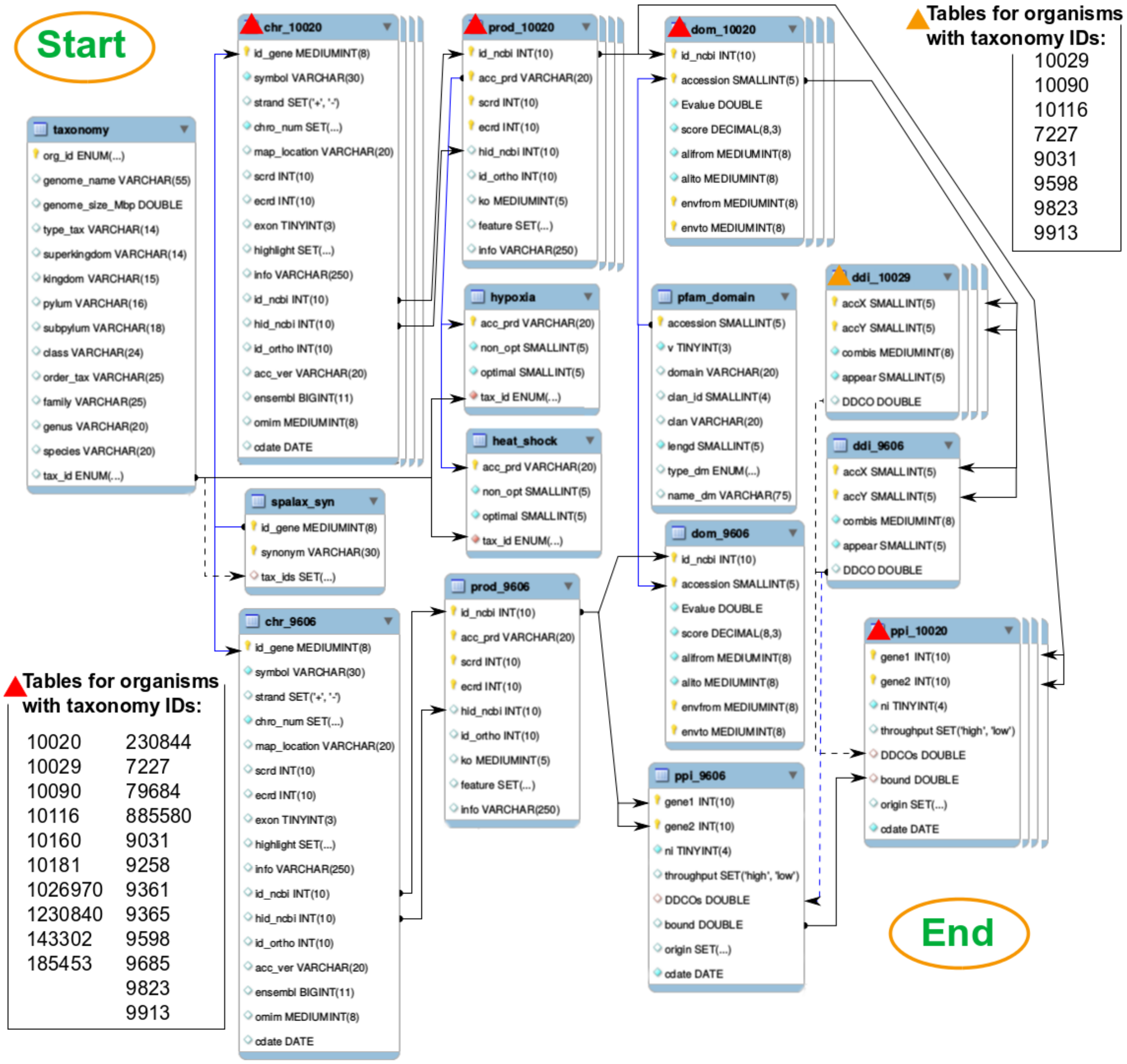

Figure S7: PASTORAL database scheme.

TABLES

Table S1. General statistics of PPI networks and related features for all organisms in PASTORAL. General information regarding PASTORAL organisms includes ecology (fossorial, subterranean, and aboveground), organism name, and organism ID, where lowercase letters represent KEGG codes and uppercase letters represents organisms not included in the KEGG database. Also included are the number of genes in total, HomoloGene (NCBI database) and the number of KO groups (total, unique). Protein-protein interaction (PPI) statistics include the number of orthologous proteins (KEGG or HomoloGene ID) that appear in PPIs and in the proteins in PPIs, the number of PPIs (real i.e., BioGrid and predicted), the intersection of real and predicted PPIs (NA – empty intersection, and in total, and in the largest linked network), the global clustering coefficient of the largest linked PPI network for organisms and the average number of interactors per protein. In addition, ecology level averages are included.
See Excel File.

Table S2. PPI network statistics for all proteins with ordered, mixed, and disordered regions. The number of interactors per protein (mean for organism and ecology, boldface) and the mean number of interactions (PPIs) are calculated. Normality test (Anderson-Darling test) of pairwise comparisons of PPIs between proteins with ordered, mixed, and disordered regions, and also pairwise comparisons of PPIs among ecologies for each protein region type, Wilcoxon rank sum test with continuity correction are calculated. Related to Table S9. Annotations for each organism are included (Taxonomy ID (NCBI), organism ID*, Organism, Name, and Ecology**) *Organisms in KEGG are coded by uppercase letters; organisms not in KEGG are coded by lowercase letters. ** aboveground, fossorial, and subterranean.
See Excel File.

Table S3. List of human RNA genes that have real (BioGrid) interactions with proteins, Gene ID, Symbol, and Refseq are included.
See Excel File.

Table S4. Comparisons of PPI features between ecologies using the Wilcoxon rank sum test with continuity correction, for full ortholog protein groups (All, A>FS, FS>A). In addition, results from the Shapiro-Wilk normality test for PPI features (average number of interactors, average number of PPIs, global/individual clustering coefficient / individual). Codon usage preference score (CUPS) is also shown. Significance at p-value <0.05 (boldface). Welch Two Sample t-test is used for normal samples. Related to Figure S1 and Figure S2.
See Excel File.

Table S5. List of hypoxia-related proteins (refseqs) belonging to KO groups.
See Excel File.

Table S6. List of heat shock proteins (refseqs) belonging to KO groups.
See Excel File.

Table S7. List of circadian proteins (refseqs) belonging to KO groups.
See Excel File.

Table S8. Codon usage preference score (CUPS) for all proteins with ordered, mixed, and disordered regions. CUPS (average for organism and ecology, boldface) and the number of interactors per protein are calculated. Normality test (Anderson-Darling test) pairwise comparison of CUPS between all proteins with ordered, mixed, and disordered regions, and also pairwise comparison of CUPS among ecologies for each protein region type, Wilcoxon rank sum test with continuity correction are calculated. Related to Table S9. Annotations for

each organism are included (Taxonomy ID (NCBI), organism ID*, Organism Name and Ecology**) *Organisms in KEGG are coded by uppercase letters; organisms not in KEGG are coded by lowercase letters. ** aboveground, fossorial, and subterranean.
See Excel File.

Table S9. Comparison of mean protein-protein interactions (PPIs)* and codon usage preference score (CUPS)** for all proteins. Within ecologies, pairwise comparison of ordered, disordered, and mixed regions (ordinality comparison); Within each region, pairwise comparisons among ecologies (ecology comparison). In general, within each ecology, proteins with mixed regions formed more PPIs and adopted more optimal codon usage preferences, on average, than did proteins with disordered regions. Proteins of subterranean animals with any region type adopted fewer optimal codon usage preferences. However, non-significant differences in the numbers of PPIs among ecologies were observed for proteins with ordered and mixed regions.

**Ecology Comparison (PPI, CUPS)**	**[O]RDER**	**[M]IXED**	**[D]ISORDER**
F vs. S	(n.s, F>S)	(S>F, F>S)	(n.s, F>S)
F vs. A	(F>A, A>F)	(n.s, A>F)	(n.s, A>F)
S vs. A	(A<S, A>S)	(S<A, A>S)	(n.s, A>S)

**Ordinality Comparison (PPI, CUPS)**	**Fossorial**	**Subterranean**	**Aboveground**
[O]vs.[M]	(M > O, M > O)	(M > O, M > O)	(M > O, n.s)
[O]vs.[D]	(O > D, n.s)	(O > D, n.s)	(O > D, O > D)
[M]vs.[D]	(M > D, M>D)	(M > D, M>D)	(M > D, M>D)

*Derived from Table S2; **Derived from Table S8; n.s non-significant at p-value > 0.05. A-aboveground, F-fossorial, S – subterranean; O - ordered regions; D – disordered, M – mixed (O and D).

Table S10. Collection of 61 intrinsically disordered proteins from the DistProt database

Organism	NCBI taxid	DisProt_ID	UniProt_ID	Disorder content, %	GeneID	Gene Symbol
D. melanogaster	7227	DP00540	Q9VPU8	100	33269	dbe
Danio rerio	7955	DP00584	A2VD23	100	386700	stm
H. sapiens	9606	DP00016	P38936	100	1026	CDKN1A
H. sapiens	9606	DP00017	P49918	100	1028	CDKN1C
H. sapiens	9606	DP00018	P46527	100	1027	CDKN1B
H. sapiens	9606	DP00028	Q13541	100	1978	EIF4EBP1
H. sapiens	9606	DP00039	P05204	100	3151	HMGN2
H. sapiens	9606	DP00040	P17096	100	3159	HMGA1
H. sapiens	9606	DP00070	P37840	100	6622	SNCA
H. sapiens	9606	DP00174	P16949	100	3925	STMN1
H. sapiens	9606	DP00214	P10451	100	6696	SPP1
H. sapiens	9606	DP00219	O60927	100	6992	PPP1R11
H. sapiens	9606	DP00221	P07476	100	3713	IVL
H. sapiens	9606	DP00332	P21815	100	3381	IBSP
H. sapiens	9606	DP00333	P27797	100	811	CALR
H. sapiens	9606	DP00357	P62328	100	7114	TMSB4X
H. sapiens	9606	DP00372	Q9NR00	100	56892	C8orf4
H. sapiens	9606	DP00510	O60356	100	26471	NUPR1
H. sapiens	9606	DP00521	O95997	100	9232	PTTG1
H. sapiens	9606	DP00543	Q14061	100	10063	COX17
H. sapiens	9606	DP00546	Q9NX55	100	25764	HYPK
H. sapiens	9606	DP00555	Q16143	100	6620	SNCB
H. sapiens	9606	DP00592	P48539	100	5121	PCP4
H. sapiens	9606	DP00630	O76070	100	6623	SNCG
H. sapiens	9606	DP00694	Q8N488	100	23429	RYBP
H. sapiens	9606	DP00930	P80723	100	10409	BASP1
H. sapiens	9606	DP00934	P61925	100	5569	PKIA
H. sapiens	9606	DP00951	P17677	100	2596	GAP43
H. sapiens	9606	DP01102	P35637	96.39	2521	FUS
H. sapiens	9606	DP01159	A6NF83	100	389493	NUPR2
H. sapiens	9606	DP01178	Q14978	100	9221	NOLC1
H. sapiens	9606	DP01293	Q13542	100	1979	EIF4EBP2
H. sapiens	9606	DP01425	Q15004	100	9768	PCLAF
H. sapiens	9606	DP01435	O60829	100	9506	PAGE4
H. sapiens	9606	DP01677	P06454	100	5757	PTMA
H. sapiens	9606	DP01876	P52926	100	8091	HMGA2
H. sapiens	9606	DP01942	P50553	100	429	ASCL1
H. sapiens	9606	DP02005	P47710	100	1446	CSN1S1
H. sapiens	9606	DP02010	Q9NRJ3	100	56477	CCL28
Sus scrofa	9823	DP00663	P81558	100	414286	MBP
Bos taurus	9913	DP00118	P05059	100	281070	CHGA
Bos taurus	9913	DP00193	P02754	100	280838	PAEP
Bos taurus	9913	DP00195	P02313	98.89	512161	HMGN2
Bos taurus	9913	DP00347	P04972	100	281977	PDE6G
Bos taurus	9913	DP00421	P07516	100	282459	PPP1R1B
Bos taurus	9913	DP00814	P01252	100	786336	PTMA

Bos taurus	9913	DP00955	P06836	100	281777	GAP43
M. musculus	10090	DP00008	Q64693	100	18985	Pou2af1
M. musculus	10090	DP00253	P26645	100	17118	Marcks
M. musculus	10090	DP00340	P62965	100	12903	Crabp1
M. musculus	10090	DP00563	Q61337	100	12015	Bad
M. musculus	10090	DP00564	Q6P8Z1	98.82	107771	Bmyc
M. musculus	10090	DP00587	Q9CQK7	100	66521	Rwdd1
M. musculus	10090	DP00645	Q91ZE9	100	171543	Bmf
M. musculus	10090	DP00661	Q9CX60	100	77889	Lbh
M. musculus	10090	DP00815	Q9DCL8	100	66849	Ppp1r2
M. musculus	10090	DP01754	Q8BHZ5	100	78412	Cyren
R. norvegicus	10116	DP00068	P60881	100	25012	Snap25
R. norvegicus	10116	DP00136	P15865	99.09	201097	Hist1h1d
R. norvegicus	10116	DP00940	Q62627	100	64513	Pawr
R. norvegicus	10116	DP01521	Q5RJL0	100	295619	Ermn

Table S11. PPI and CUPS calculated for subterranean proteins derived from the DistProt database. Related to Table S10.
See Excel File.

Table S12. PPI and CUPS calculated for fossorial proteins derived from the DistProt database. Related to Table S10.
See Excel File.

Table S13. PPI and CUPS calculated for aboveground proteins derived from the DistProt database. Related to Table S10.
See Excel File.

References

61 Vallabhajosyula, R. R., Chakravarti, D., Lutfeali, S., Ray, A. & Raval, A. Identifying hubs in protein interaction networks. *PLoS One* **4**, e5344, doi:10.1371/journal.pone.0005344 (2009).

62 Gottlieb, A., Frenkel-Morgenstern, M., Safro, M. & Horn, D. Common peptides study of aminoacyl-tRNA synthetases. *PLoS One* **6**, e20361, doi:10.1371/journal.pone.0020361 (2011).

63 Chang, X., Xu, T., Li, Y. & Wang, K. Dynamic modular architecture of protein-protein interaction networks beyond the dichotomy of 'date' and 'party' hubs. *Sci Rep* **3**, 1691, doi:10.1038/srep01691 (2013).

64 Brito, A. F. & Pinney, J. W. Protein-Protein Interactions in Virus-Host Systems. *Front Microbiol* **8**, 1557, doi:10.3389/fmicb.2017.01557 (2017).

65 Agarwal, S., Deane, C. M., Porter, M. A. & Jones, N. S. Revisiting date and party hubs: novel approaches to role assignment in protein interaction networks. *PLoS Comput Biol* **6**, e1000817, doi:10.1371/journal.pcbi.1000817 (2010).

66 Reddy, P. *et al.* Molecular analysis of the period locus in Drosophila melanogaster and identification of a transcript involved in biological rhythms. *Cell* **38**, 701 - 710 (1984).

67 Crews, S. T., Thomas, J. B. & Goodman, C. S. The Drosophila single-minded gene encodes a nuclear protein with sequence similarity to the per gene product. *Cell* **52**, 143 - 151 (1988).

68 Furukawa-Hibi, Y., Nagai, T., Yun, J. & Yamada, K. Stress increases DNA methylation of the neuronal PAS domain 4 (Npas4) gene. *Neuroreport* **26**, 827-832, doi:10.1097/WNR.0000000000000430 (2015).

69 Kim, S. H. *et al.* Molecular characteristics and induction profiles of hypoxia-inducible factor-1α and other basic helix-loop-helix and Per-Arnt-Sim domain-containing proteins identified in a carcinogenic liver fluke Clonorchis sinensis. *Parasitology*, 1-11, doi:10.1017/S0031182018001245 (2018).

70 Kolonko, M. *et al.* Intrinsic Disorder of the C-Terminal Domain of Drosophila Methoprene-Tolerant Protein. *PLoS One* **11**, e0162950, doi:10.1371/journal.pone.0162950 (2016).

71 Tang, X., Shao, J. & Qin, X. Crystal structure of the PAS domain of the hEAG potassium channel. *Acta Crystallogr F Struct Biol Commun* **72**, 578-585, doi:10.1107/S2053230X16009419 (2016).

72 Salvi, M. *et al.* Sensory domain contraction in histidine kinase CitA triggers transmembrane signaling in the membrane-bound sensor. *Proc Natl Acad Sci U S A* **114**, 3115-3120, doi:10.1073/pnas.1620286114 (2017).

73 Greb-Markiewicz, B., Zarębski, M. & Ożyhar, A. Multiple sequences orchestrate subcellular trafficking of neuronal PAS domain-containing protein 4 (NPAS4). *J Biol Chem* **293**, 11255-11270, doi:10.1074/jbc.RA118.001812 (2018).

74 Lesne, E. *et al.* Coiled-Coil Antagonism Regulates Activity of Venus Flytrap-Domain-Containing Sensor Kinases of the BvgS Family. *MBio* **9**, doi:10.1128/mBio.02052-17 (2018).

75 Sarkar, J., Miller, D. P., Oliver, L. D. & Marconi, R. T. The Treponema denticola PAS Domain-Containing Histidine Kinase Hpk2 Is a Heme Binding Sensor of Oxygen Levels. *J Bacteriol* **200**, doi:10.1128/JB.00116-18 (2018).

76 Dioum, E. M. *et al.* NPAS2: A Gas-Responsive Transcription Factor. *Science* **298**, 2385-2387 (2002).

77 Koudo, R. *et al.* Spectroscopic characterization of the isolated heme-bound PAS-B domain of neuronal PAS domain protein 2 associated with circadian rhythms. *FEBS Journal* **272**, 4153-4162 (2005).

78 Cai, Z. *et al.* Adaptive Transcriptome Profiling of Subterranean Zokor, Myospalax baileyi, to High- Altitude Stresses in Tibet. *Scientific Reports* **8**, 4671 (2018)
